# Abortion Surveillance — United States, 2018

**DOI:** 10.15585/mmwr.ss6907a1

**Published:** 2020-11-27

**Authors:** Katherine Kortsmit, Tara C. Jatlaoui, Michele G. Mandel, Jennifer A. Reeves, Titilope Oduyebo, Emily Petersen, Maura K. Whiteman

**Affiliations:** ^1^Division of Reproductive Health, National Center for Chronic Disease Prevention and Health Promotion, CDC; ^2^Oak Ridge Institute for Science and Education

## Abstract

**Problem/Condition:**

CDC conducts abortion surveillance to document the number and characteristics of women obtaining legal induced abortions and number of abortion-related deaths in the United States.

**Period Covered:**

2018.

**Description of System:**

Each year, CDC requests abortion data from the central health agencies for 50 states, the District of Columbia, and New York City. For 2018, 49 reporting areas voluntarily provided aggregate abortion data to CDC. Of these, 48 reporting areas provided data each year during 2009–2018. Census and natality data were used to calculate abortion rates (number of abortions per 1,000 women aged 15–44 years) and ratios (number of abortions per 1,000 live births), respectively. Abortion-related deaths from 2017 were assessed as part of CDC’s Pregnancy Mortality Surveillance System (PMSS).

**Results:**

A total of 619,591 abortions for 2018 were reported to CDC from 49 reporting areas. Among 48 reporting areas with data each year during 2009–2018, in 2018, a total of 614,820 abortions were reported, the abortion rate was 11.3 abortions per 1,000 women aged 15–44 years, and the abortion ratio was 189 abortions per 1,000 live births. From 2017 to 2018, the total number of abortions and abortion rate increased 1% (from 609,095 total abortions and from 11.2 abortions per 1,000 women aged 15–44 years, respectively), and the abortion ratio increased 2% (from 185 abortions per 1,000 live births). From 2009 to 2018, the total number of reported abortions, abortion rate, and abortion ratio decreased 22% (from 786,621), 24% (from 14.9 abortions per 1,000 women aged 15–44 years), and 16% (from 224 abortions per 1,000 live births), respectively.

In 2018, women in their 20s accounted for more than half of abortions (57.7%). In 2018 and during 2009–2018, women aged 20–24 and 25–29 years accounted for the highest percentages of abortions; in 2018, they accounted for 28.3% and 29.4% of abortions, respectively, and had the highest abortion rates (19.1 and 18.5 per 1,000 women aged 20–24 and 25–29 years, respectively). By contrast, adolescents aged <15 years and women aged ≥40 years accounted for the lowest percentages of abortions (0.2% and 3.6%, respectively) and had the lowest abortion rates (0.4 and 2.6 per 1,000 women aged <15 and ≥40 years, respectively). However, abortion ratios in 2018 and throughout 2009–2018 were highest among adolescents (aged ≤19 years) and lowest among women aged 25–39 years. Abortion rates decreased from 2009 to 2018 for all women, regardless of age. The decrease in abortion rate was highest among adolescents compared with women in any other age group. From 2009 to 2013, the abortion rates decreased for all age groups and from 2014 to 2018, the abortion rates decreased for all age groups, except for women aged 30–34 years and those aged ≥40 years. In addition, from 2017 to 2018, abortion rates did not change or decreased among women aged ≤24 and ≥40 years; however, the abortion rate increased among women aged 25–39 years. Abortion ratios also decreased from 2009 to 2018 among all women, except adolescents aged <15 years. The decrease in abortion ratio was highest among women aged ≥40 years compared with women in any other age group. The abortion ratio decreased for all age groups from 2009 to 2013; however, from 2014 to 2018, abortion ratios only decreased for women aged ≥35 years. From 2017 to 2018, abortion ratios increased for all age groups, except women aged ≥40 years.

In 2018, approximately three fourths (77.7%) of abortions were performed at ≤9 weeks’ gestation, and nearly all (92.2%) were performed at ≤13 weeks’ gestation. In 2018, and during 2009–2018, the percentage of abortions performed at >13 weeks’ gestation remained consistently low (≤9.0%). In 2018, the highest proportion of abortions were performed by surgical abortion at ≤13 weeks’ gestation (52.1%), followed by early medical abortion at ≤9 weeks’ gestation (38.6%), surgical abortion at >13 weeks’ gestation (7.8%), and medical abortion at >9 weeks’ gestation (1.4%); all other methods were uncommon (<0.1%). Among those that were eligible (≤9 weeks’ gestation), 50.0% of abortions were early medical abortions. In 2017, the most recent year for which PMSS data were reviewed for pregnancy-related deaths, two women were identified to have died as a result of complications from legal induced abortion.

**Interpretation:**

Among the 48 areas that reported data continuously during 2009–2018, decreases were observed during 2009–2017 in the total number, rate, and ratio of reported abortions, and these decreases resulted in historic lows for this period for all three measures. These decreases were followed by 1%–2% increases across all measures from 2017 to 2018.

**Public Health Action:**

The data in this report can help program planners and policymakers identify groups of women with the highest rates of abortion. Unintended pregnancy is a major contributor to induced abortion. Increasing access to and use of effective contraception can reduce unintended pregnancies and further reduce the number of abortions performed in the United States.

## Introduction

This report summarizes data on legal induced abortions for 2018 that were provided voluntarily to CDC by the central health agencies of 49 reporting areas (47 states, the District of Columbia, and New York City, excluding California, Maryland, and New Hampshire) and comparisons over time for the 48 reporting areas that reported each year during 2009–2018 (47 states and New York City). A summary of data for the 49 reporting areas that provided data voluntarily to CDC for 2017 is available (Supplementary Tables; https://stacks.cdc.gov/view/cdc/96608). This report also summarizes abortion-related deaths reported voluntarily to CDC for 2017 as part of the Pregnancy Mortality Surveillance System (PMSS).

Since 1969, CDC has conducted abortion surveillance to document the number and characteristics of women obtaining legal induced abortions in the United States (*1*). After nationwide legalization of abortion in 1973, the total number, rate (number of abortions per 1,000 women aged 15–44 years), and ratio (number of abortions per 1,000 live births) of reported abortions increased rapidly, reaching the highest levels in the 1980s, before decreasing at a slow yet steady pace (*2*–*4*). During 2006–2008, a break occurred in the previously sustained pattern of decrease (*5*–*8*), although this break has been followed in subsequent years by even greater decreases (*9*–*19*). Nonetheless, throughout the years, abortion incidence continues to vary across subpopulations (*20*–*26*). Continued surveillance is needed to monitor changes in abortion incidence in the United States.

## Methods

### Description of the Surveillance System

Each year, CDC requests aggregated data from the central health agencies of the 50 states, the District of Columbia, and New York City to document the number and characteristics of women obtaining legal induced abortions in the United States. This report contains data voluntarily reported to CDC as of February 29, 2020. For the purpose of surveillance, a legal induced abortion* is defined as an intervention performed within the limits of state law by a licensed clinician (e.g., a physician, nurse-midwife, nurse practitioner, or physician assistant) intended to terminate a suspected or known intrauterine pregnancy and that does not result in a live birth.

In most states and jurisdictions, collection of abortion data is facilitated by a legal requirement for hospitals, facilities, and physicians to report abortions to a central health agency (*27*); however, reporting is not complete in all areas, including in some areas with reporting requirements (*28*). Central health agencies voluntarily report aggregate abortion data to CDC. Because the reporting of abortion data to CDC is voluntary, many reporting areas have developed their own data collection forms and therefore do not collect or provide all of the information requested by CDC. As a result, the level of detail reported by CDC on the characteristics of women obtaining abortions varies from year to year and by reporting area (*18*). To encourage uniform collection of data, CDC has collaborated with the National Association for Public Health Statistics and Information Systems to develop reporting standards and provide technical guidance for vital statistics personnel who collect and summarize abortion data within the United States.

### Variables and Categorization of Data

Each year, CDC sends a suggested template to central health agencies in the United States for compilation of aggregated abortion data. Aggregate abortion numbers, without individual-level records, are requested for the following variables:

- Age group in years of women obtaining legal induced abortions (<15, 15–19 by individual year, 20–24, 25–29, 30–34, 35–39, or ≥40)
- Gestational age of pregnancy in completed weeks at the time of abortion (≤6, 7–20 by individual week, or ≥21)
- Race (Black, White, or other [including Asian, Pacific Islander, other races, and multiple races]), ethnicity (Hispanic or non-Hispanic), and race by ethnicity
- Method type (surgical abortion,^†^ intrauterine instillation, medical [nonsurgical] abortion, or hysterectomy/hysterotomy)
- Marital status (married [including currently married or separated] or unmarried [including never married, widowed, or divorced])
- Number of previous live births (0, 1, 2, 3, or ≥4)
- Number of previous induced abortions (0, 1, 2, or ≥3)
- Residence (the state, jurisdiction, territory, or foreign country in which the woman obtaining the abortion lived, or, if additional details are unavailable, in-reporting area versus out-of-reporting area)

In addition, the template provided by CDC requests that aggregate numbers for certain variables be cross-tabulated by a second variable. The cross-tabulations presented in this report include weeks of gestation separately by method type, by women’s age group, and by race/ethnicity.

Beginning with 2014 data, instead of reporting clinician’s estimates of gestational age or estimates of gestational age based on last menstrual period, some areas have reported “probable postfertilization age,” “clinician’s estimate of gestation based on date of conception,” and “probable gestational age” to CDC. To make data reported as postfertilization age consistent with data collection practices for gestational age recommended by CDC’s National Center for Health Statistics (*29*), 2 weeks were added to probable postfertilization age. This method was used to account for time after last menstrual period until ovulation in a standard 28-day cycle because fertilization occurs around the time of ovulation (*30*). No modifications were made to data reported as clinician’s estimate of gestation based on date of conception or data reported as probable gestational age.

In this report, medical and surgical abortions are further categorized by gestational age when available. Early medical abortion is defined as the administration of medications (typically mifepristone followed by misoprostol) to induce an abortion at ≤9 completed weeks’ gestation,^§^ consistent with the current Food and Drug Administration (FDA) labeling for mifepristone (implemented in 2016) (*31*). Medications (typically serial prostaglandins, sometimes administered after mifepristone) may also be used to induce an abortion at >9 weeks’ gestation. Surgical abortions are categorized as having been performed at ≤13 weeks’ gestation or at >13 weeks’ gestation because of differences in surgical technique at these gestational ages (*32*). Finally, because intrauterine instillations cannot be performed early in gestation, abortions reported to have been performed by intrauterine instillation at ≤12 weeks’ gestation are excluded from calculation of the percentage of abortions by known method type and are grouped with unknown type.^¶^

### Measures of Abortion

Four measures of abortion are presented in this report: 1) the number of abortions in a given population, 2) the percentage of abortions among women by selected characteristics, 3) the abortion rate (number of abortions per 1,000 women within a given population), and 4) the abortion ratio (number of abortions per 1,000 live births within a given population). Abortion rates adjust for differences in population size. Abortion ratios measure the relative number of pregnancies in a population that end in abortion compared with live birth.

U.S. Census Bureau estimates of the resident female population were used as the denominator for calculating abortion rates (*33*–*42*). Overall abortion rates were calculated from the population of women aged 15–44 years living in the reporting areas that provided data. For adolescents aged <15 years, abortion rates were calculated using the number of adolescents aged 13–14 years; for women aged ≥40 years, abortion rates were calculated using the number of women aged 40–44 years. For the calculation of abortion ratios, live birth data were obtained from CDC natality files and included births to women of all ages living in the reporting areas that provided abortion data (*43*–*45*). For calculation of the total abortion rates and total ratios only, women with unknown data on selected characteristics (e.g., age, race/ethnicity, and marital status) were distributed according to the distribution of abortions among women with known information on the characteristic. For calculation of totals only, abortions for women with an unknown gestational age of pregnancy but known method type were distributed according to the distribution of abortions among women with known information on method type by gestational age to the following categories: surgical, ≤13 weeks’ gestation; surgical, >13 weeks’ gestation; medical, ≤9 weeks’ gestation; and medical, >9 weeks’ gestation.

### Data Presentation and Analysis

This report provides aggregate and reporting area–specific abortion numbers, rates, and ratios for the 49 areas that reported to CDC for 2018, which excluded California, Maryland, and New Hampshire. In addition, this report describes characteristics of women who obtained abortions in 2018. The data in this report are presented by the reporting area in which the abortions were performed. Overall abortion rates and ratios are not presented for reporting areas with <20 total reported abortions because calculations are considered statistically unstable (*46*). Wyoming, which reported <20 abortions, was only included in total abortions overall and was excluded from all subsequent analyses.

The completeness and quality of data received varies by year and by variable; this report only describes the characteristics of women obtaining abortions in reporting areas that met CDC reporting standards (i.e., reported at least 20 abortions overall, provided data categorized in accordance with requested variables, and had <15% unknown values for a given characteristic). Cells with a value in the range of 1–4 or cells that would allow for calculation of these values have been suppressed in this report to maintain confidentiality.

Trends in the number, rate, and ratio of reported abortions and annual data are presented for the 48 areas that reported data every year during 2009–2018. The percentage change in abortion measures from the most recent past year (2017 to 2018) and during the 10-year period of analysis (2009 to 2018) were calculated for these 48 reporting areas.

Trends were also reported for abortions by age group of women obtaining abortions and by weeks of gestation. Annual data are presented for areas that met reporting standards every year during 2009–2018; the percentage change was calculated from the beginning to the end of the 10-year period of analysis (2009–2018), from the beginning to the end of the first and second halves of this period (2009–2013 and 2014–2018), and from the most recent past year (2017 to 2018). Consistent with previous reports, key findings for trends are presented to highlight observed changes over time and differences between groups. However, no statistical testing was performed. Comparisons do not imply statistical significance, and lack of comment regarding the difference between values does not imply that no statistically significant difference exists.

To calculate trends for early medical abortions (≤9 completed weeks’ gestation), areas were included if they met reporting standards and if they specifically included medical abortion as a method on their reporting form for the years needed for 10-year, 5-year, and 1-year percentage change calculations (2009 to 2018, 2009 to 2013, 2014 to 2018, and 2017 to 2018). These data are reported to monitor any changes in clinical practice that might have occurred with the accumulation of evidence on the safety and effectiveness of medical abortion past 63 days of gestation (≤8 completed weeks) (*47*), changes in professional practice guidelines published in 2013 and 2014 (*48*,*49*), and the 2016 FDA extension of the gestational age limit for the use of mifepristone for early medical abortion from 63 days to 70 days (≤9 completed weeks’ gestation) (*50*).

Data from some reporting areas are not included in trends if the data did not meet reporting standards every year during 2009–2018 (for overall, age, and gestational age trend analyses) or if data did not meet reporting standards for selected years of comparison (for early medical abortion trend analysis). As a result, aggregate measures for 2018 in trend analyses might differ from the point estimates reported for 2018.

### Abortion Mortality

CDC has reported data on abortion-related deaths periodically since information on abortion mortality first was included in the 1972 abortion surveillance report (*18*,*51*). An abortion-related death is defined as a death resulting from a direct complication of an abortion (legal or illegal), an indirect complication caused by a chain of events initiated by an abortion, or an aggravation of a preexisting condition by the physiologic or psychologic effects of abortion (*52*). An abortion is categorized as legal when it is performed by a licensed clinician within the limits of state law.

Since 1987, CDC has monitored abortion-related deaths through PMSS (*53*,*54*). Sources of data to identify abortion-related deaths have included state vital records; media reports, including computerized searches of full-text newspaper and other print media databases; and individual case reports by public health agencies, including maternal mortality review committees, health care providers and provider organizations, private citizens, and citizen groups. For each death that is possibly related to abortion, CDC requests clinical records and autopsy reports. Two medical epidemiologists independently review these reports to determine the cause of death and whether the death was abortion related. Discrepancies are discussed and resolved by consensus. Each death is categorized by abortion type as legal induced, illegal induced, spontaneous, or unknown type.

This report provides PMSS data on induced abortion-related deaths that occurred in 2017, the most recent year for which PMSS data are available. Data on induced abortion-related deaths that occurred during 1972–2015 have been published (*12*–*15*,*17*,*18*,*54*). For 1998–2017, abortion surveillance data reported to CDC cannot be used alone to calculate national legal induced abortion case-fatality rates (number of legal induced abortion-related deaths per 100,000 reported legal induced abortions in the United States) because eight states** did not report abortion data every year during this period. Thus, denominator data for calculation of national legal induced abortion case-fatality rates were obtained from a published report by the Guttmacher Institute that includes estimated total numbers of abortions in the United States from a national survey of abortion-providing facilities (*19*). Because rates determined on the basis of a numerator of <20 deaths are unstable (*46*), national legal induced abortion case-fatality rates were calculated for consecutive 5-year periods during 1973–2017.

## Results

### Total Abortions Reported to CDC by Occurrence 

Among the 49 reporting areas that provided data for 2018, a total of 619,591 abortions were reported. Of these abortions, 614,820 (99.2%) were from 48 reporting areas that provided data every year for 2009–2018. In 2018, these continuously reporting areas had an abortion rate of 11.3 abortions per 1,000 women aged 15–44 years and an abortion ratio of 189 abortions per 1,000 live births (Table 1). In 2017, the total number, rate, and ratio of reported abortions decreased to historic lows for the period of analysis for all three measures. From 2017 to 2018, the total number of reported abortions and abortion rate increased 1% (from 609,095 to 614,820 total abortions and from 11.2 to 11.3 abortions per 1,000 women aged 15–44 years), and the abortion ratio increased 2% (from 185 to 189 abortions per 1,000 live births). From 2009 to 2018, the total number of reported abortions decreased 22% (from 786,621), the abortion rate decreased 24% (from 14.9 abortions per 1,000 women aged 15–44 years), and the abortion ratio decreased 16% (from 224 abortions per 1,000 live births) (Figure).

**TABLE 1:** Number, percentage, rate,* and ratio^†^ of reported abortions — selected reporting areas, United States, 2009–2018

Year	Selected reporting areas^§^	Continuously reporting areas^¶^		
	No.	No. (%)**	Rate	Ratio
2009	789,217^††^	786,621 (99.7)	14.9	224
2010	765,651	762,755 (99.6)	14.4	225
2011	730,322	727,554 (99.6)	13.7	217
2012	699,202	696,587 (99.6)	13.1	208
2013	664,435	661,874 (99.6)	12.4	198
2014	652,639	649,849 (99.6)	12.1	192
2015	638,169	636,902 (99.8)	11.8	188
2016	623,471	623,471 (100.0)	11.6	186
2017	612,719	609,095 (99.4)	11.2	185
2018	619,591	614,820 (99.2)	11.3	189

* Number of abortions per 1,­­000 women aged 15–44 years.

^†^ Number of abortions per 1,000 live births.

^§^ For each given year, excludes reporting areas that did not report that year’s abortion numbers to CDC: California (2009–2018), District of Columbia (2016), Maryland (2009–2018), and New Hampshire (2009–2018).

^¶^ For all years, excludes reporting areas that did not report abortion numbers every year during the period of analysis (2009–2018): California, District of Columbia, Maryland, and New Hampshire.

** Abortions from areas that reported every year during 2009–2018 as a percentage of all reported abortions for a given year.

^††^ This number is greater than reported in the 2009 report because of numbers subsequently provided by Delaware (**Source:** Pazol K, Creanga AA, Zane SB, Burley KD, Jamieson DJ. Abortion surveillance—United States, 2009. MMWR Surveill Summ 2012;61[No. SS-8]).

**FIGURE:**
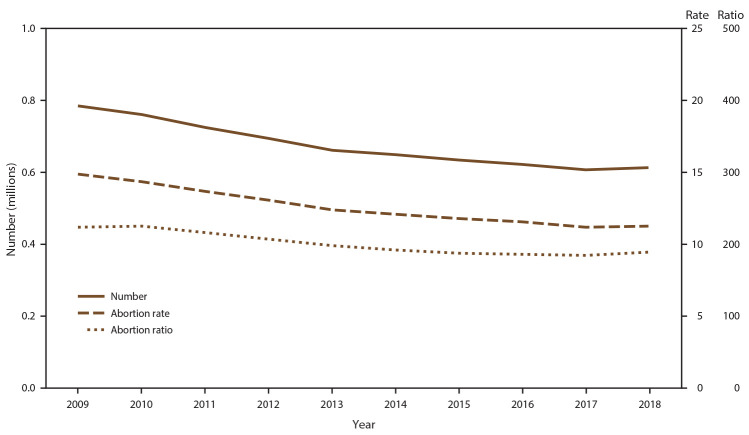
Number, rate,* and ratio^†^ of abortions performed, by year — selected reporting areas,^§^ United States, 2009–2018 * Number of abortions per 1,000 women aged 15–44 years. ^†^ Number of abortions per 1,000 live births. ^§^ Data are for 48 reporting areas; excludes California, District of Columbia, Maryland, and New Hampshire.

In 2018, a considerable range existed in abortion rates by reporting area of occurrence (from 2.4 to 26.8 abortions per 1,000 women aged 15–44 years in South Dakota and New York City) and abortion ratios (from 32 to 518 abortions per 1,000 live births in South Dakota and the District of Columbia)^††^ (Table 2). The percentage of abortions obtained by out-of-state residents also varied among reporting areas (from 0.4% in Arizona to 65.4% in the District of Columbia). Overall, 9.3% of abortions were reported to CDC with unknown residence.

**TABLE 2:** Number, rate,* and ratio^†^ of reported abortions, by reporting area of occurrence and percentage of abortions obtained by out-of-state residents^§^ — United States, 2018^¶^

State/Area	Abortions reported by area of occurrence**			Abortions obtained by out-of-state residents
	No.	Rate	Ratio	No. (%)
Alabama	6,484	6.8	112	1,029 (15.9)
Alaska	1,283	8.8	127	17 (1.3)
Arizona	12,438	9.0	154	48 (0.4)
Arkansas	3,069	5.3	83	321 (10.5)
Colorado	8,975	7.7	143	1,032 (11.5)
Connecticut	9,294	13.9	268	423 (4.6)
Delaware	1,740	9.7	164	236 (13.6)
District of Columbia^††^	4,771	25.3	518	3,119 (65.4)
Florida	70,239	18.1	317	2,653 (3.8)
Georgia	33,918	15.7	269	5,558 (16.4)
Hawaii	2,121	8.0	125	31 (1.5)
Idaho	1,257	3.7	59	48 (3.8)
Illinois	42,441	16.9	293	5,668 (13.4)
Indiana	8,037	6.2	98	774 (9.6)
Iowa	2,849	4.8	75	302 (10.6)
Kansas	6,972	12.4	192	3,498 (50.2)
Kentucky	3,203	3.8	59	489 (15.3)
Louisiana	8,097	8.7	136	1,315 (16.2)
Maine	1,949	8.4	158	81 (4.2)
Massachusetts	18,256	13.1	264	575 (3.1)
Michigan	26,716	14.2	243	1,158 (4.3)
Minnesota	9,910	9.2	147	1,014 (10.2)
Mississippi	3,005	5.1	81	317 (10.5)
Missouri	2,910	2.5	40	265 (9.1)
Montana	1,674	8.7	145	170 (10.2)
Nebraska	2,078	5.6	82	246 (11.8)
Nevada	8,819	14.8	247	456 (5.2)
New Jersey^§§^	22,936	13.6	227	1,304 (5.7)
New Mexico	3,847	9.7	167	853 (22.2)
New York	77,447	19.8	342	7,514 (9.7)
New York City	49,759	26.8	457	4,308 (8.7)
New York State	27,688	13.5	236	3,206 (11.6)
North Carolina	27,581	13.5	232	4,996 (18.1)
North Dakota	1,141	7.7	107	309 (27.1)
Ohio	20,425	9.2	151	1,210 (5.9)
Oklahoma	4,990	6.5	100	368 (7.4)
Oregon	8,735	10.6	207	828 (9.5)
Pennsylvania	30,364	12.7	224	2,124 (7.0)
Rhode Island	2,817	13.5	268	382 (13.6)
South Carolina	4,646	4.8	82	227 (4.9)
South Dakota	382	2.4	32	85 (22.3)
Tennessee	10,880	8.2	135	2,063 (19.0)
Texas	55,140	9.2	146	1,234 (2.2)
Utah	3,082	4.5	65	187 (6.1)
Vermont	1,204	10.5	222	215 (17.9)
Virginia	16,474	9.8	165	935 (5.7)
Washington	17,264	11.5	201	934 (5.4)
West Virginia	1,507	4.7	83	208 (13.8)
Wisconsin	6,224	5.7	97	182 (2.9)
**Total**	**619,591**	**NA**	**NA**	**NA**

**Abbreviation: **NA = not applicable.

* Number of abortions per 1,000 women aged 15–44 years.

^†^ Number of abortions per 1,000 live births.

^§^ Additional details on the reporting area in which abortions were provided, cross-tabulated by the state of residence, are available at https://www.cdc.gov/reproductivehealth/data_stats/Abortion.htm.

^¶^ Data from 48 reporting areas; excludes four reporting areas (California, Maryland, New Hampshire, and Wyoming) that did not report or did not meet reporting standards.

** The total abortions include those with known and unknown residence status.

^††^ Reporting to the central health agency is not required.

^§§^ Reporting to the central health agency is not required. Data are requested from hospitals and licensed ambulatory care facilities only.

### Age Group, Race/Ethnicity, and Marital Status

Among the 48 areas that reported abortion numbers by women’s age for 2018, women in their 20s accounted for the majority (57.7%) of abortions and had the highest abortion rates (19.1 and 18.5 abortions per 1,000 women aged 20–24 and 25–29 years, respectively) (Table 3). Women in the youngest (<15 years) and oldest (≥40 years) age groups accounted for the smallest percentages of abortions (0.2% and 3.6%, respectively) and had the lowest abortion rates (0.4 and 2.6 abortions per 1,000 women aged <15 and ≥40 years, respectively). In contrast, abortion ratios in 2018 were lowest among women aged 25–39 years (126–189 per 1,000 live births).

**TABLE 3:** Reported abortions, by known age group and reporting area of occurrence — selected reporting areas,* United States, 2018

State/Area	Age group (yrs)							Total abortions reported by known age
	<15	15–19	20–24	25–29	30–34	35–39	≥40	
	No. (%)^†^	No. (%)	No. (%)	No. (%)	No. (%)	No. (%)	No. (%)	No. (% of all reported abortions)^§^
Alabama	24 (0.4)	634 (9.8)	1,997 (30.8)	1,956 (30.2)	1,104 (17.0)	585 (9.0)	181 (2.8)	**6,481 (100.0)**
Alaska	—^¶^	154 (12.0)	365 (28.4)	355 (27.7)	233 (18.2)	131 (10.2)	—	**1,283 (100.0)**
Arizona	28 (0.2)	1,126 (9.1)	3,787 (30.4)	3,471 (27.9)	2,178 (17.5)	1,380 (11.1)	467 (3.8)	**12,437 (100.0)**
Arkansas	17 (0.6)	290 (9.4)	974 (31.7)	879 (28.6)	507 (16.5)	315 (10.3)	87 (2.8)	**3,069 (100.0)**
Colorado	26 (0.3)	850 (9.5)	2,619 (29.2)	2,566 (28.6)	1,663 (18.6)	913 (10.2)	327 (3.6)	**8,964 (99.9)**
Connecticut	24 (0.3)	845 (9.3)	2,489 (27.3)	2,697 (29.6)	1,738 (19.1)	1,003 (11.0)	305 (3.4)	**9,101 (97.9)**
Delaware	—	201 (11.6)	516 (29.7)	473 (27.2)	314 (18.0)	175 (10.1)	—	**1,740 (100.0)**
District of Columbia**	13 (0.3)	437 (9.2)	1,349 (28.3)	1,461 (30.6)	882 (18.5)	460 (9.6)	168 (3.5)	**4,770 (100.0)**
Florida	101 (0.1)	5,370 (7.7)	18,999 (27.1)	20,643 (29.5)	14,207 (20.3)	7,899 (11.3)	2,863 (4.1)	**70,082 (99.8)**
Georgia	74 (0.2)	2,717 (8.0)	9,523 (28.1)	10,454 (30.8)	6,443 (19.0)	3,554 (10.5)	1,153 (3.4)	**33,918 (100.0)**
Hawaii	—	200 (9.4)	627 (29.6)	567 (26.7)	406 (19.1)	227 (10.7)	—	**2,121 (100.0)**
Idaho	—	138 (11.0)	411 (32.7)	329 (26.2)	200 (15.9)	140 (11.1)	—	**1,256 (99.9)**
Illinois^††^	94 (0.3)	3,223 (8.8)	10,702 (29.3)	11,043 (30.2)	6,632 (18.1)	3,729 (10.2)	1,164 (3.2)	**36,587 (99.7)**
Indiana	22 (0.3)	787 (9.8)	2,513 (31.3)	2,291 (28.5)	1,381 (17.2)	771 (9.6)	272 (3.4)	**8,037 (100.0)**
Iowa	7 (0.2)	283 (10.0)	822 (28.9)	785 (27.6)	557 (19.6)	282 (9.9)	107 (3.8)	**2,843 (99.8)**
Kansas	15 (0.2)	647 (9.3)	2,221 (31.9)	1,942 (27.9)	1,152 (16.5)	741 (10.6)	254 (3.6)	**6,972 (100.0)**
Kentucky	14 (0.4)	287 (9.0)	933 (29.1)	917 (28.6)	601 (18.8)	338 (10.6)	113 (3.5)	**3,203 (100.0)**
Louisiana	18 (0.2)	721 (8.9)	2,400 (29.6)	2,399 (29.6)	1,508 (18.6)	786 (9.7)	265 (3.3)	**8,097 (100.0)**
Maine	—	194 (10.0)	581 (29.8)	527 (27.1)	337 (17.3)	228 (11.7)	—	**1,947 (99.9)**
Massachusetts	25 (0.1)	1,256 (6.9)	4,678 (25.6)	5,283 (28.9)	3,943 (21.6)	2,276 (12.5)	793 (4.3)	**18,254 (100.0)**
Michigan	54 (0.2)	2,265 (8.5)	7,719 (29.0)	8,651 (32.5)	4,652 (17.5)	2,450 (9.2)	814 (3.1)	**26,605 (99.6)**
Minnesota	16 (0.2)	793 (8.0)	2,713 (27.4)	2,775 (28.0)	2,037 (20.6)	1,193 (12.0)	383 (3.9)	**9,910 (100.0)**
Mississippi	13 (0.4)	285 (9.5)	899 (29.9)	932 (31.0)	530 (17.6)	276 (9.2)	70 (2.3)	**3,005 (100.0)**
Missouri	14 (0.5)	286 (9.8)	861 (29.6)	861 (29.6)	542 (18.6)	272 (9.4)	73 (2.5)	**2,909 (100.0)**
Montana	—	183 (11.0)	479 (28.7)	468 (28.0)	307 (18.4)	172 (10.3)	—	**1,671 (99.8)**
Nebraska	9 (0.4)	220 (10.6)	663 (31.9)	546 (26.3)	360 (17.3)	206 (9.9)	74 (3.6)	**2,078 (100.0)**
Nevada	17 (0.2)	713 (8.3)	2,356 (27.5)	2,444 (28.5)	1,704 (19.9)	982 (11.5)	353 (4.1)	**8,569 (97.2)**
New Jersey^§§^	69 (0.3)	2,023 (8.8)	6,167 (26.9)	6,779 (29.6)	4,479 (19.5)	2,507 (10.9)	907 (4.0)	**22,931 (100.0)**
New Mexico	9 (0.2)	502 (13.9)	1,066 (29.4)	932 (25.7)	651 (18.0)	328 (9.1)	134 (3.7)	**3,622 (94.2)**
New York	168 (0.2)	6,843 (8.9)	20,723 (26.8)	21,988 (28.5)	15,272 (19.8)	9,035 (11.7)	3,221 (4.2)	**77,250 (99.7)**
New York City	108 (0.2)	3,984 (8.0)	12,833 (25.8)	14,259 (28.7)	10,238 (20.6)	6,047 (12.2)	2,288 (4.6)	**49,757 (100.0)**
New York State	60 (0.2)	2,859 (10.4)	7,890 (28.7)	7,729 (28.1)	5,034 (18.3)	2,988 (10.9)	933 (3.4)	**27,493 (99.3)**
North Carolina	72 (0.3)	2,240 (8.4)	7,550 (28.3)	8,198 (30.7)	4,987 (18.7)	2,794 (10.5)	840 (3.1)	**26,681 (96.7)**
North Dakota	—	103 (9.0)	329 (28.8)	332 (29.1)	215 (18.8)	116 (10.2)	—	**1,141 (100.0)**
Ohio	54 (0.3)	1,884 (9.2)	6,128 (30.0)	6,206 (30.4)	3,639 (17.8)	1,912 (9.4)	602 (2.9)	**20,425 (100.0)**
Oklahoma	25 (0.5)	471 (9.4)	1,507 (30.2)	1,474 (29.6)	904 (18.1)	469 (9.4)	135 (2.7)	**4,985 (99.9)**
Oregon	12 (0.1)	809 (9.3)	2,462 (28.2)	2,415 (27.7)	1,650 (18.9)	1,028 (11.8)	353 (4.0)	**8,729 (99.9)**
Pennsylvania	72 (0.2)	2,529 (8.3)	8,644 (28.5)	9,252 (30.5)	5,753 (18.9)	3,191 (10.5)	923 (3.0)	**30,364 (100.0)**
Rhode Island	—	218 (7.8)	827 (29.6)	814 (29.1)	526 (18.8)	294 (10.5)	—	**2,797 (99.3)**
South Carolina	14 (0.3)	449 (9.7)	1,360 (29.3)	1,343 (28.9)	814 (17.5)	510 (11.0)	156 (3.4)	**4,646 (100.0)**
South Dakota	0 (0.0)	41 (10.7)	112 (29.3)	109 (28.5)	79 (20.7)	31 (8.1)	10 (2.6)	**382 (100.0)**
Tennessee	22 (0.2)	972 (9.0)	3,241 (29.9)	3,320 (30.6)	1,981 (18.3)	1,048 (9.7)	270 (2.5)	**10,854 (99.8)**
Texas	112 (0.2)	4,856 (8.8)	16,187 (29.4)	15,790 (28.6)	10,322 (18.7)	5,935 (10.8)	1,938 (3.5)	**55,140 (100.0)**
Utah	6 (0.2)	412 (13.4)	946 (30.8)	769 (25.0)	511 (16.6)	324 (10.6)	102 (3.3)	**3,070 (99.6)**
Vermont	—	101 (8.4)	358 (29.8)	318 (26.5)	220 (18.3)	146 (12.1)	—	**1,202 (99.8)**
Virginia	16 (0.1)	1,196 (7.3)	4,499 (27.3)	4,927 (29.9)	3,226 (19.6)	1,938 (11.8)	649 (3.9)	**16,451 (99.9)**
Washington	36 (0.2)	1,725 (10.0)	4,722 (27.4)	4,738 (27.5)	3,288 (19.1)	2,033 (11.8)	706 (4.1)	**17,248 (99.9)**
West Virginia	5 (0.3)	140 (9.3)	459 (30.5)	430 (28.5)	266 (17.7)	155 (10.3)	52 (3.5)	**1,507 (100.0)**
Wisconsin^††^	17 (0.3)	561 (9.3)	1,839 (30.4)	1,771 (29.3)	1,080 (17.9)	615 (10.2)	159 (2.6)	**6,042 (100.0)**
**Total**	**1,362 (0.2)**	**53,180 (8.7)**	**173,322 (28.3)**	**179,620 (29.4)**	**115,981 (19.0)**	**65,893 (10.8)**	**22,018 (3.6)**	**611,376 (99.6)^¶¶^**
Abortion rate***	**0.4**	**6.0**	**19.1**	**18.5**	**12.6**	**7.2**	**2.6**	**—**
**Abortion ratio^†††^**	**872**	**334**	**271**	**189**	**126**	**142**	**218**	**—**

* Data from 48 reporting areas; excludes four reporting areas (California, Maryland, New Hampshire, and Wyoming) that did not report, did not report by age, or did not meet reporting standards.

^†^ Percentages for the individual component categories might not add to 100% because of rounding.

^§^ Percentage is calculated as the number of abortions reported by known age divided by the sum of abortions reported by known and unknown age. Values ≥99.95% are rounded to 100.0%.

^¶^ Cells with a value in the range of 1–4 or cells that would allow for calculation of these small values have been suppressed.

** Reporting to the central health agency is not required.

^††^ Includes residents only.

^§§^ Reporting to the central health agency is not required. Data are requested from hospitals and licensed ambulatory care facilities only.

^¶¶^ Percentage based on a total of 613,681 abortions reported among the areas that met reporting standards for age.

*** Number of abortions obtained by women in a given age group per 1,000 women in that same age group. Adolescents aged 13–14 years were used as the denominator for the group of adolescents aged <15 years, and women aged 40–44 years were used as the denominator for the group of women aged ≥40 years. For the total abortion rate only, abortions for women of unknown age were distributed according to the distribution of abortions among women of known age.

^†††^ Number of abortions obtained by women in a given age group per 1,000 live births to women in that same age group. For the total abortion ratio only, abortions for women of unknown age were distributed according to the distribution of abortions among women of known age.

Among the 44 reporting areas that provided data each year by women’s age for 2009–2018, this pattern across age groups was stable, with the majority of abortions and the highest abortion rates occurring among women aged 20–29 years and the lowest percentages of abortions and abortion rates occurring among women in the youngest and oldest age groups (Table 4). From 2009 to 2018, abortion rates decreased among all age groups, although the decreases for adolescents (64% and 55% for adolescents aged <15 and 15–19 years, respectively) were greater than the decreases for women in all older age groups. From 2009 to 2013, the abortion rates decreased for all age groups, and from 2014 to 2018, the abortion rates decreased for all age groups except women aged 30–34 years and ≥40 years. From 2017 to 2018, abortion rates did not change or decreased among women aged ≤24 and ≥40 years; however, the abortion rate increased among women aged 25–39 years. During 2009–2018, abortion ratios decreased among women in all age groups, except for adolescents aged <15 years. The abortion ratio decreased for all age groups from 2009 to 2013; however, from 2014 to 2018, abortion ratios only decreased for women aged ≥35 years. From 2017 to 2018, abortion ratios increased for all age groups, except women aged ≥40 years.

**TABLE 4:** Reported abortions, by known age group and year — selected reporting areas,* United States, 2009–2018

Age group (yrs)	Year										% change			
	2009	2010	2011	2012	2013	2014	2015	2016	2017	2018	2009 to 2013	2014 to 2018	2017 to 2018	2009 to 2018
**Reported abortions by known age (%)**														
<15	0.5	0.5	0.4	0.4	0.3	0.3	0.3	0.3	0.2	0.2	−40.0	−33.3	0.0	−60.0
15–19	15.5	14.6	13.5	12.2	11.4	10.4	9.8	9.4	9.1	8.8	−26.5	−15.4	−3.3	−43.2
20–24	32.7	32.9	32.9	32.8	32.7	32.1	31.1	30.0	29.3	28.5	0.0	−11.2	−2.7	−12.8
25–29	24.4	24.5	24.9	25.4	25.9	26.8	27.6	28.5	29.0	29.4	6.1	9.7	1.4	20.5
30–34	14.8	15.3	15.8	16.4	16.8	17.2	17.7	18.0	18.3	18.8	13.5	9.3	2.7	27.0
35–39	8.8	8.9	8.9	9.1	9.2	9.7	10.0	10.3	10.5	10.7	4.5	10.3	1.9	21.6
≥40	3.3	3.4	3.6	3.7	3.6	3.6	3.6	3.6	3.6	3.5	9.1	−2.8	−2.8	6.1
**Abortion rate^†^**														
<15	1.1	1.0	0.9	0.8	0.6	0.5	0.5	0.4	0.4	0.4	−45.5	−20.0	0.0	−63.6
15–19	12.8	11.7	10.5	9.2	8.2	7.3	6.7	6.2	5.9	5.8	−35.9	−20.5	−1.7	−54.7
20–24	27.7	26.8	25.0	23.3	21.9	20.9	19.9	19.1	18.3	18.2	−20.9	−12.9	−0.5	−34.3
25–29	20.7	20.2	19.4	18.9	18.2	18.1	17.9	17.8	17.3	17.6	−12.1	−2.8	1.7	−15.0
30–34	13.4	13.2	12.7	12.4	11.8	11.7	11.7	11.6	11.5	11.9	−11.9	1.7	3.5	−11.2
35–39	7.6	7.6	7.5	7.3	7.0	7.1	7.0	6.9	6.7	6.8	−7.9	−4.2	1.5	−10.5
≥40	2.8	2.8	2.8	2.8	2.5	2.5	2.5	2.5	2.5	2.5	−10.7	0.0	0.0	−10.7
**Abortion ratio^§^**														
<15	832	848	839	804	791	745	700	733	777	853	−4.9	14.5	9.8	2.5
15–19	328	332	325	304	299	291	289	296	301	318	−8.8	9.3	5.6	−3.0
20–24	281	290	284	272	262	256	250	250	249	256	−6.8	0.0	2.8	−8.9
25–29	183	184	178	174	168	166	167	169	171	178	−8.2	7.2	4.1	−2.7
30–34	138	138	132	128	121	116	115	113	114	119	−12.3	2.6	4.4	−13.8
35–39	172	171	165	158	147	145	140	136	134	135	−14.5	−6.9	0.7	−21.5
≥40	275	273	275	269	245	239	228	220	211	206	−10.9	−13.8	−2.4	−25.1
**Total (no.)**	**695,952**	**675,732**	**643,628**	**614,570**	**582,260**	**569,100**	**556,221**	**544,663**	**528,130**	**533,375**	**—**	**—**	**—**	**—**

* Data from 44 reporting areas; excludes eight reporting areas (California, District of Columbia, Florida, Maine, Maryland, New Hampshire, Vermont, and Wyoming) that did not report, did not report by age, or did not meet reporting standards for ≥1 year. By year, these reporting areas represent 87%–99% of all abortions reported by age to CDC during 2009–2018.

^†^ Number of abortions obtained by women in a given age group per 1,000 women in that same age group. Adolescents aged 13–14 years were used as the denominator for the group of adolescents aged <15 years, and women aged 40–44 years were used as the denominator for the group of women aged ≥40 years. Women aged 15–44 years were used as the denominator for the overall rate. For the total abortion rate only, abortions for women of unknown age were distributed according to the distribution of abortions among women of known age.

^§^ Number of abortions obtained by women in a given age group per 1,000 live births to women in that same age group. For the total abortion ratio only, abortions for women of unknown age were distributed according to the distribution of abortions among women of known age.

Among the 46 areas^§§^ that reported women’s age by individual year among adolescents for 2018, adolescents aged 18–19 years accounted for the majority (69.7%) of adolescent abortions and had the highest adolescent abortion rates (8.6 and 12.2 abortions per 1,000 adolescents aged 18 and 19 years, respectively). Adolescents aged <15 years accounted for the smallest percentage of adolescent abortions (2.5%) and had the lowest adolescent abortion rate (0.4 abortions per 1,000 adolescents aged 13–14 years). In 2018, the abortion ratio for adolescents was highest among adolescents aged <15 years (833 abortions per 1,000 live births) and was lowest among adolescents aged ≥17 years (336, 346, and 284 abortions per 1,000 live births among adolescents aged 17, 18, and 19 years, respectively).

Among the 31 areas that reported race/ethnicity data for 2018, non-Hispanic White women and non-Hispanic Black women accounted for the largest percentages of all abortions (38.7% and 33.6%, respectively), and Hispanic women and non-Hispanic women in the other race category accounted for smaller percentages (20.0% and 7.7%, respectively) (Table 5). Non-Hispanic White women had the lowest abortion rate (6.3 abortions per 1,000 women) and ratio (110 abortions per 1,000 live births), and non-Hispanic Black women had the highest abortion rate (21.2 abortions per 1,000 women) and ratio (335 abortions per 1,000 live births).

**TABLE 5:** Reported abortions, by known race/ethnicity and reporting area of occurrence — selected reporting areas,* United States, 2018

State/Area	Non-Hispanic			Hispanic	Total abortions reported by known race/ethnicity
	White	Black	Other		No. (% of all reported abortions)^§^
	No. (%)^†^	No. (%)	No. (%)	No. (%)	
Alabama	1,986 (30.7)	3,981 (61.5)	170 (2.6)	332 (5.1)	**6,469 (99.8)**
Alaska	603 (51.1)	77 (6.5)	447 (37.9)	53 (4.5)	**1,180 (92.0)**
Arizona	4,828 (40.6)	1,237 (10.4)	1,109 (9.3)	4,714 (39.7)	**11,888 (95.6)**
Arkansas	1,380 (45.2)	1,318 (43.2)	158 (5.2)	198 (6.5)	**3,054 (99.5)**
Connecticut	3,351 (38.8)	2,809 (32.5)	449 (5.2)	2,027 (23.5)	**8,636 (92.9)**
Delaware	742 (42.9)	722 (41.8)	71 (4.1)	193 (11.2)	**1,728 (99.3)**
District of Columbia^¶^	883 (19.4)	2,498 (55.0)	529 (11.6)	634 (14.0)	**4,544 (95.2)**
Florida	21,215 (32.8)	21,898 (33.9)	2,814 (4.4)	18,684 (28.9)	**64,611 (92.0)**
Idaho	881 (73.0)	37 (3.1)	54 (4.5)	235 (19.5)	**1,207 (96.0)**
Indiana	4,326 (54.8)	2,422 (30.7)	463 (5.9)	687 (8.7)	**7,898 (98.3)**
Kansas	3,930 (56.5)	1,649 (23.7)	497 (7.1)	882 (12.7)	**6,958 (99.8)**
Kentucky	1,873 (58.5)	973 (30.4)	128 (4.0)	228 (7.1)	**3,202 (100.0)**
Michigan	10,731 (40.9)	13,215 (50.4)	1,309 (5.0)	985 (3.8)	**26,240 (98.2)**
Minnesota	4,488 (49.4)	2,421 (26.7)	1,396 (15.4)	774 (8.5)	**9,079 (91.6)**
Mississippi	655 (21.9)	2,147 (71.9)	104 (3.5)	79 (2.6)	**2,985 (99.3)**
Missouri	1,231 (42.6)	1,375 (47.5)	218 (7.5)	69 (2.4)	**2,893 (99.4)**
Montana	1,401 (83.7)	28 (1.7)	155 (9.3)	90 (5.4)	**1,674 (100.0)**
Nevada	3,236 (38.5)	1,466 (17.5)	1,058 (12.6)	2,635 (31.4)	**8,395 (95.2)**
New Jersey**	6,565 (30.8)	6,696 (31.4)	3,341 (15.7)	4,732 (22.2)	**21,334 (93.0)**
North Carolina	8,348 (32.4)	12,487 (48.4)	1,922 (7.5)	3,031 (11.8)	**25,788 (93.5)**
Ohio	8,028 (46.0)	7,183 (41.2)	1,226 (7.0)	1,014 (5.8)	**17,451 (85.4)**
Oregon	4,954 (65.5)	449 (5.9)	844 (11.2)	1,314 (17.4)	**7,561 (86.6)**
South Carolina	2,181 (47.0)	1,873 (40.4)	234 (5.0)	351 (7.6)	**4,639 (99.8)**
South Dakota	237 (62.2)	51 (13.4)	56 (14.7)	37 (9.7)	**381 (99.7)**
Tennessee	4,417 (41.6)	5,258 (49.5)	331 (3.1)	620 (5.8)	**10,626 (97.7)**
Texas^††^	14,990 (27.2)	14,759 (26.8)	3,948 (7.2)	21,408 (38.8)	**55,105 (99.9)**
Utah	2,077 (70.8)	123 (4.2)	240 (8.2)	494 (16.8)	**2,934 (95.2)**
Vermont	1,052 (89.8)	28 (2.4)	53 (4.5)	39 (3.3)	**1,172 (97.3)**
Virginia	4,943 (34.6)	6,500 (45.4)	1,361 (9.5)	1,500 (10.5)	**14,304 (86.8)**
Washington	8,468 (57.7)	1,798 (12.2)	2,266 (15.4)	2,149 (14.6)	**14,681 (85.0)**
West Virginia	1,328 (88.1)	148 (9.8)	24 (1.6)	7 (0.5)	**1,507 (100.0)**
**Total**	**135,328 (38.7)**	**117,626 (33.6)**	**26,975 (7.7)**	**70,195 (20.0)**	**350,124 (94.1)^§§^**
**Abortion rate^¶¶^**	**6.3**	**21.2**	**11.9**	**10.9**	**—**
Abortion ratio***	**110**	**335**	**213**	**158**	**—**

* Data from 31 reporting areas; excludes 21 reporting areas (California, Colorado, Georgia, Hawaii, Illinois, Iowa, Louisiana, Maine, Maryland, Massachusetts, Nebraska, New Hampshire, New Mexico, New York City, New York State, North Dakota, Oklahoma, Pennsylvania, Rhode Island, Wisconsin, and Wyoming) that did not report, did not report by race/ethnicity, or did not meet reporting standards.

^†^ Percentages for the individual component categories might not add to 100% because of rounding.

^§^ Percentage is calculated as the number of abortions reported by known race/ethnicity divided by the sum of abortions reported by known and unknown race/ethnicity. Values ≥99.95% are rounded to 100.0%.

^¶^ Reporting to the central health agency is not required.

**Reporting to the central health agency is not required. Data are requested from hospitals and licensed ambulatory care facilities only.

^††^ Reporting form contains only one question for race/ethnicity; therefore, abortions reported for women of White, Black, and other races (Asian and Native American) are not explicitly identified as non-Hispanic.

^§§^ Percentage is based on a total of 372,077 abortions reported among the areas that met reporting standards for race/ethnicity.

^¶¶^ Number of abortions obtained by women in a given racial/ethnic group per 1,000 women in that same racial/ethnic group. For the total abortion rate only, abortions for women of unknown race/ethnicity were distributed according to the distribution of abortions among women of known race/ethnicity.

*** Number of abortions obtained by women in a given racial/ethnic group per 1,000 live births to women in that same racial/ethnic group. For the total abortion ratio only, abortions for women of unknown race/ethnicity were distributed according to the distribution of abortions among women of known race/ethnicity.

Among the 42 areas that reported by marital status for 2018, 14.8% of women who obtained an abortion were married, and 85.2% were unmarried (Table 6). The abortion ratio was 44 abortions per 1,000 live births for married women and 378 abortions per 1,000 live births for unmarried women.

**TABLE 6:** Reported abortions, by known marital status and reporting area of occurrence — selected reporting areas,* United States, 2018

State/Area	Marital status		Total abortions reported by known marital status
	Married	Unmarried	
	No. (%)^†^	No. (%)	No. (% of all reported abortions)^§^
Alabama	711 (11.0)	5,772 (89.0)	**6,483 (100.0)**
Alaska	274 (21.9)	977 (78.1)	**1,251 (97.5)**
Arizona	1,728 (13.9)	10,710 (86.1)	**12,438 (100.0)**
Arkansas	398 (13.0)	2,657 (87.0)	**3,055 (99.5)**
Colorado	1,393 (18.2)	6,254 (81.8)	**7,647 (85.2)**
Connecticut	886 (10.7)	7,392 (89.3)	**8,278 (89.1)**
Delaware	214 (12.3)	1,526 (87.7)	**1,740 (100.0)**
Florida	9,682 (15.8)	51,725 (84.2)	**61,407 (87.4)**
Georgia	4,103 (13.9)	25,461 (86.1)	**29,564 (87.2)**
Idaho	229 (20.8)	874 (79.2)	**1,103 (87.7)**
Illinois^¶^	3,578 (10.3)	31,052 (89.7)	**34,630 (94.3)**
Indiana	1,178 (14.7)	6,858 (85.3)	**8,036 (100.0)**
Iowa	474 (16.6)	2,373 (83.4)	**2,847 (99.9)**
Kansas	1,096 (15.7)	5,871 (84.3)	**6,967 (99.9)**
Kentucky	479 (15.0)	2,724 (85.0)	**3,203 (100.0)**
Louisiana	868 (11.1)	6,939 (88.9)	**7,807 (96.4)**
Maine	310 (16.6)	1,560 (83.4)	**1,870 (95.9)**
Massachusetts	2,757 (17.1)	13,362 (82.9)	**16,119 (88.3)**
Michigan	2,806 (10.7)	23,355 (89.3)	**26,161 (97.9)**
Minnesota	1,560 (16.4)	7,936 (83.6)	**9,496 (95.8)**
Mississippi	283 (9.5)	2,686 (90.5)	**2,969 (98.8)**
Missouri	401 (14.6)	2,348 (85.4)	**2,749 (94.5)**
Montana	289 (17.3)	1,382 (82.7)	**1,671 (99.8)**
Nebraska	322 (15.6)	1,740 (84.4)	**2,062 (99.2)**
New Jersey**	2,720 (12.0)	19,922 (88.0)	**22,642 (98.7)**
New Mexico	559 (15.3)	3,089 (84.7)	**3,648 (94.8)**
New York City	7,888 (18.4)	34,943 (81.6)	**42,831 (86.1)**
North Carolina	3,821 (15.3)	21,142 (84.7)	**24,963 (90.5)**
North Dakota	183 (16.1)	957 (83.9)	**1,140 (99.9)**
Ohio	2,670 (14.5)	15,703 (85.5)	**18,373 (90.0)**
Oklahoma	932 (18.7)	4,052 (81.3)	**4,984 (99.9)**
Oregon	1,666 (20.6)	6,411 (79.4)	**8,077 (92.5)**
Pennsylvania	3,521 (11.6)	26,832 (88.4)	**30,353 (100.0)**
South Carolina	648 (14.1)	3,956 (85.9)	**4,604 (99.1)**
South Dakota	71 (18.6)	311 (81.4)	**382 (100.0)**
Tennessee	1,448 (13.6)	9,199 (86.4)	**10,647 (97.9)**
Texas	9,656 (17.5)	45,484 (82.5)	**55,140 (100.0)**
Utah	761 (24.9)	2,297 (75.1)	**3,058 (99.2)**
Vermont	227 (20.4)	887 (79.6)	**1,114 (92.5)**
Virginia	2,492 (15.1)	13,982 (84.9)	**16,474 (100.0)**
West Virginia	283 (18.8)	1,223 (81.2)	**1,506 (99.9)**
Wisconsin^¶^	830 (13.8)	5,198 (86.2)	**6,028 (99.8)**
**Total**	**76,395 (14.8)**	**439,122 (85.2)**	**515,517 (93.7)^††^**
**Abortion ratio^§§^**	**44**	**378**	**—**

* Data from 42 reporting areas; excludes 10 areas (California, District of Columbia, Hawaii, Maryland, Nevada, New Hampshire, New York State, Rhode Island, Washington, and Wyoming) that did not report, did not report by marital status, or did not meet reporting standards.

^†^ Percentages for the individual component categories might not add to 100% because of rounding.

^§^ Percentage is calculated as the number of abortions reported by known marital status divided by the sum of abortions reported by known and unknown marital status. Values ≥99.95% are rounded to 100.0%.

^¶^ Includes residents only.

** Reporting to the central health agency is not required. Data are requested from hospitals and licensed ambulatory care facilities only.

^††^ Percentage is based on a total of 550,201 abortions reported among the areas that met reporting standards for marital status.

^§§^ Number of abortions obtained by women by marital status per 1,000 live births to women of the same marital status. For the total abortion ratio only, abortions for women of unknown marital status were distributed according to the distribution of abortions among women of known marital status.

### Previous Live Births and Abortions

Data from the 43 areas that reported the number of previous live births for women who obtained abortions in 2018 indicate that 40.7%, 24.8%, 19.8%, and 14.7% of these women had zero, one, two, or three or more previous live births, respectively (Table 7). Data from the 40 areas that reported the number of previous abortions for women who obtained abortions in 2018 indicate that the majority (59.9%) had previously had no abortions, 23.9% had previously had one abortion, 9.9% had previously had two abortions, and 6.4% had previously had three or more abortions (Table 8).

**TABLE 7:** Reported abortions, by known number of previous live births and reporting area of occurrence — selected reporting areas,* United States, 2018

State/Area	No. of previous live births					Total abortions reported by known number of previous live births
	0	1	2	3	≥4	
	No. (%)^†^	No. (%)	No. (%)	No. (%)	No. (%)	No. (% of all reported abortions)^§^
Alabama	2,258 (34.8)	1,826 (28.2)	1,434 (22.1)	630 (9.7)	336 (5.2)	**6,484 (100.0)**
Alaska	588 (45.8)	246 (19.2)	208 (16.2)	112 (8.7)	129 (10.1)	**1,283 (100.0)**
Arizona	5,472 (44.3)	2,633 (21.3)	2,332 (18.9)	1,151 (9.3)	773 (6.3)	**12,361 (99.4)**
Arkansas	1,068 (34.8)	858 (28.0)	652 (21.2)	313 (10.2)	178 (5.8)	**3,069 (100.0)**
Colorado	4,921 (56.7)	1,619 (18.6)	1,225 (14.1)	570 (6.6)	347 (4.0)	**8,682 (96.7)**
Connecticut	4,097 (44.1)	2,230 (24.0)	1,778 (19.1)	774 (8.3)	415 (4.5)	**9,294 (100.0)**
Delaware	763 (43.9)	421 (24.2)	313 (18.0)	150 (8.6)	92 (5.3)	**1,739 (99.9)**
Florida	26,311 (37.5)	18,256 (26.0)	14,979 (21.3)	6,417 (9.1)	4,276 (6.1)	**70,239 (100.0)**
Georgia	15,141 (44.6)	7,702 (22.7)	6,159 (18.2)	2,834 (8.4)	2,075 (6.1)	**33,911 (100.0)**
Hawaii	1,143 (55.1)	385 (18.6)	300 (14.5)	160 (7.7)	85 (4.1)	**2,073 (97.7)**
Idaho	578 (46.4)	269 (21.6)	213 (17.1)	119 (9.6)	66 (5.3)	**1,245 (99.0)**
Indiana	3,158 (39.3)	1,959 (24.4)	1,623 (20.2)	840 (10.5)	457 (5.7)	**8,037 (100.0)**
Iowa	1,160 (40.7)	649 (22.8)	603 (21.2)	272 (9.5)	165 (5.8)	**2,849 (100.0)**
Kansas	2,882 (41.3)	1,684 (24.2)	1,337 (19.2)	647 (9.3)	422 (6.1)	**6,972 (100.0)**
Kentucky	1,224 (38.2)	825 (25.8)	670 (20.9)	311 (9.7)	173 (5.4)	**3,203 (100.0)**
Louisiana	2,665 (33.0)	2,221 (27.5)	1,806 (22.3)	854 (10.6)	537 (6.6)	**8,083 (99.8)**
Massachusetts	7,656 (47.5)	3,730 (23.2)	2,895 (18.0)	1,204 (7.5)	620 (3.8)	**16,105 (88.2)**
Michigan^¶^	9,278 (34.7)	7,161 (26.8)	5,816 (21.8)	2,745 (10.3)	1,709 (6.4)	**26,709 (100.0)**
Minnesota	3,885 (39.3)	2,242 (22.7)	1,982 (20.0)	1,025 (10.4)	757 (7.7)	**9,891 (99.8)**
Mississippi	956 (31.8)	852 (28.4)	655 (21.8)	349 (11.6)	191 (6.4)	**3,003 (99.9)**
Missouri	1,091 (37.7)	725 (25.1)	613 (21.2)	259 (9.0)	205 (7.1)	**2,893 (99.4)**
Montana	795 (47.5)	343 (20.5)	301 (18.0)	150 (9.0)	85 (5.1)	**1,674 (100.0)**
Nebraska	800 (38.6)	507 (24.4)	434 (20.9)	215 (10.4)	119 (5.7)	**2,075 (99.9)**
Nevada	3,781 (42.9)	1,963 (22.3)	1,708 (19.4)	825 (9.4)	542 (6.1)	**8,819 (100.0)**
New Jersey**	8,998 (39.5)	6,926 (30.4)	4,220 (18.5)	1,939 (8.5)	682 (3.0)	**22,765 (99.3)**
New Mexico	1,442 (41.0)	789 (22.5)	626 (17.8)	360 (10.2)	297 (8.5)	**3,514 (91.3)**
New York City	21,240 (46.7)	11,469 (25.2)	8,054 (17.7)	3,042 (6.7)	1,703 (3.7)	**45,508 (91.5)**
North Carolina	9,246 (36.6)	6,244 (24.7)	5,188 (20.6)	2,587 (10.2)	1,980 (7.8)	**25,245 (91.5)**
North Dakota	442 (38.7)	241 (21.1)	222 (19.5)	137 (12.0)	99 (8.7)	**1,141 (100.0)**
Ohio	7,294 (36.0)	5,288 (26.1)	4,319 (21.3)	2,053 (10.1)	1,293 (6.4)	**20,247 (99.1)**
Oklahoma	1,385 (31.7)	1,185 (27.1)	1,015 (23.2)	496 (11.4)	288 (6.6)	**4,369 (87.6)**
Oregon	4,329 (49.9)	1,846 (21.3)	1,461 (16.8)	661 (7.6)	380 (4.4)	**8,677 (99.3)**
Pennsylvania	11,533 (38.0)	7,976 (26.3)	6,149 (20.3)	2,925 (9.6)	1,781 (5.9)	**30,364 (100.0)**
Rhode Island	1,692 (60.3)	476 (17.0)	421 (15.0)	153 (5.5)	65 (2.3)	**2,807 (99.6)**
South Carolina	1,938 (41.7)	1,180 (25.4)	935 (20.1)	370 (8.0)	223 (4.8)	**4,646 (100.0)**
South Dakota	133 (34.8)	96 (25.1)	93 (24.3)	38 (9.9)	22 (5.8)	**382 (100.0)**
Tennessee	3,839 (35.9)	2,831 (26.5)	2,305 (21.6)	1,061 (9.9)	659 (6.2)	**10,695 (98.3)**
Texas	21,732 (39.4)	13,287 (24.1)	11,444 (20.8)	5,415 (9.8)	3,258 (5.9)	**55,136 (100.0)**
Utah	1,778 (57.7)	518 (16.8)	447 (14.5)	184 (6.0)	155 (5.0)	**3,082 (100.0)**
Vermont	610 (50.7)	236 (19.6)	236 (19.6)	93 (7.7)	29 (2.4)	**1,204 (100.0)**
Virginia	6,075 (36.9)	4,265 (25.9)	3,602 (21.9)	1,618 (9.8)	914 (5.5)	**16,474 (100.0)**
Washington	7,971 (46.2)	3,857 (22.4)	3,182 (18.5)	1,408 (8.2)	820 (4.8)	**17,238 (99.8)**
West Virginia	450 (29.9)	462 (30.7)	374 (24.8)	144 (9.6)	77 (5.1)	**1,507 (100.0)**
**Total**	**213,798 (40.7)**	**130,478 (24.8)**	**104,329 (19.8)**	**47,610 (9.1)**	**29,479 (5.6)**	**525,694 (98.0)^††^**

* Data from 43 reporting areas; excludes nine areas (California, District of Columbia, Illinois, Maine, Maryland, New Hampshire, New York State, Wisconsin, and Wyoming) that did not report, did not report by number of previous live births, or did not meet reporting standards.

^†^ Percentages for the individual component categories might not add to 100% because of rounding.

^§^ Percentage is calculated as the number of abortions reported by known number of previous live births, divided by the sum of abortions reported by known and unknown number of previous live births. Values ≥99.95% are rounded to 100.0%.

^¶^ Recorded as the number of previous pregnancies carried to term.

** Reporting to the central health agency is not required. Data are requested from hospitals and licensed ambulatory care facilities only.

^††^ Percentage is based on a total of 536,518 abortions reported among the areas that met reporting standards for the number of previous live births.

**TABLE 8:** Reported abortions, by known number of previous induced abortions and reporting area of occurrence — selected reporting areas,* United States, 2018

State/Area	Number of previous induced abortions				Total abortions reported by known number of previous induced abortions
	0	1	2	≥3	
	No. (%)^†^	No. (%)	No. (%)	No. (%)	No. (% of all reported abortions)^§^
Alabama	4,234 (65.3)	1,479 (22.8)	507 (7.8)	264 (4.1)	**6,484 (100.0)**
Alaska	840 (65.5)	282 (22.0)	109 (8.5)	52 (4.1)	**1,283 (100.0)**
Arizona	7,949 (64.6)	3,005 (24.4)	923 (7.5)	437 (3.5)	**12,314 (99.0)**
Arkansas	1,941 (63.2)	672 (21.9)	233 (7.6)	223 (7.3)	**3,069 (100.0)**
Colorado	6,025 (68.9)	1,874 (21.4)	607 (6.9)	235 (2.7)	**8,741 (97.4)**
Connecticut	5,020 (54.0)	2,224 (23.9)	1,069 (11.5)	981 (10.6)	**9,294 (100.0)**
Delaware	1,101 (63.5)	406 (23.4)	151 (8.7)	76 (4.4)	**1,734 (99.7)**
Florida	39,335 (56.0)	17,286 (24.6)	7,654 (10.9)	5,964 (8.5)	**70,239 (100.0)**
Georgia	21,686 (64.0)	7,482 (22.1)	3,019 (8.9)	1,722 (5.1)	**33,909 (100.0)**
Hawaii	1,310 (62.5)	461 (22.0)	199 (9.5)	126 (6.0)	**2,096 (98.8)**
Idaho	956 (76.8)	201 (16.1)	64 (5.1)	24 (1.9)	**1,245 (99.0)**
Indiana	5,560 (69.2)	1,685 (21.0)	554 (6.9)	238 (3.0)	**8,037 (100.0)**
Iowa	1,923 (67.5)	593 (20.8)	211 (7.4)	121 (4.2)	**2,848 (100.0)**
Kansas	4,721 (67.7)	1,440 (20.7)	529 (7.6)	282 (4.0)	**6,972 (100.0)**
Kentucky	2,074 (64.8)	706 (22.0)	245 (7.6)	178 (5.6)	**3,203 (100.0)**
Louisiana	5,028 (62.2)	2,017 (25.0)	750 (9.3)	289 (3.6)	**8,084 (99.8)**
Massachusetts	9,218 (51.9)	5,011 (28.2)	2,146 (12.1)	1,394 (7.8)	**17,769 (97.3)**
Michigan	13,674 (51.2)	6,880 (25.8)	3,523 (13.2)	2,632 (9.9)	**26,709 (100.0)**
Minnesota	5,971 (60.3)	2,203 (22.3)	963 (9.7)	758 (7.7)	**9,895 (99.8)**
Mississippi	2,002 (66.7)	678 (22.6)	231 (7.7)	92 (3.1)	**3,003 (99.9)**
Missouri	1,773 (61.3)	690 (23.9)	290 (10.0)	138 (4.8)	**2,891 (99.3)**
Montana	628 (37.5)	690 (41.2)	236 (14.1)	120 (7.2)	**1,674 (100.0)**
Nebraska	1,452 (69.9)	423 (20.4)	131 (6.3)	72 (3.5)	**2,078 (100.0)**
Nevada	4,969 (56.3)	2,325 (26.4)	943 (10.7)	582 (6.6)	**8,819 (100.0)**
New Jersey^¶^	15,580 (68.0)	3,988 (17.4)	1,823 (8.0)	1,533 (6.7)	**22,924 (99.9)**
North Carolina	14,893 (59.0)	6,396 (25.3)	2,659 (10.5)	1,292 (5.1)	**25,240 (91.5)**
North Dakota	722 (63.3)	250 (21.9)	106 (9.3)	63 (5.5)	**1,141 (100.0)**
Ohio	12,131 (60.0)	4,871 (24.1)	1,997 (9.9)	1,211 (6.0)	**20,210 (98.9)**
Oregon	5,376 (61.9)	1,937 (22.3)	811 (9.3)	556 (6.4)	**8,680 (99.4)**
Pennsylvania	15,880 (52.3)	7,703 (25.4)	3,798 (12.5)	2,983 (9.8)	**30,364 (100.0)**
Rhode Island	2,008 (71.6)	457 (16.3)	223 (8.0)	115 (4.1)	**2,803 (99.5)**
South Carolina	2,605 (56.1)	1,172 (25.2)	509 (11.0)	360 (7.7)	**4,646 (100.0)**
South Dakota	267 (69.9)	75 (19.6)	24 (6.3)	16 (4.2)	**382 (100.0)**
Tennessee	6,801 (63.2)	2,659 (24.7)	892 (8.3)	406 (3.8)	**10,758 (98.9)**
Texas	34,508 (62.6)	13,548 (24.6)	4,765 (8.6)	2,315 (4.2)	**55,136 (100.0)**
Utah	2,528 (82.0)	398 (12.9)	106 (3.4)	50 (1.6)	**3,082 (100.0)**
Vermont	764 (63.5)	272 (22.6)	116 (9.6)	52 (4.3)	**1,204 (100.0)**
Virginia	9,117 (55.3)	4,312 (26.2)	1,909 (11.6)	1,136 (6.9)	**16,474 (100.0)**
Washington	10,354 (60.1)	4,035 (23.4)	1,629 (9.5)	1,211 (7.0)	**17,229 (99.8)**
West Virginia	876 (58.2)	405 (26.9)	147 (9.8)	78 (5.2)	**1,506 (99.9)**
**Total **	**283,800 (59.9)**	**113,191 (23.9)**	**46,801 (9.9)**	**30,377 (6.4)**	474,169 (99.2)**

* Data from 40 reporting areas; excludes 12 areas (California, District of Columbia, Illinois, Maine, Maryland, New Hampshire, New Mexico, New York City, New York State, Oklahoma, Wisconsin, and Wyoming) that did not report, did not report by the number of previous induced abortions, or did not meet reporting standards.

^†^ Percentages for the individual component categories might not add to 100% because of rounding.

^§^ Percentage is calculated as the number of abortions reported by known number of previous induced abortions divided by the sum of abortions reported by known and unknown number of previous induced abortions. Values ≥99.95% are rounded to 100.0%.

^¶^ Reporting to the central health agency is not required. Data are requested from hospitals and licensed ambulatory care facilities only.

** Percentage is based on a total of 477,922 abortions reported among the areas that met reporting standards for the number of previous induced abortions.

### Weeks of Gestation and Method Type

Among the 42 areas that reported gestational age^¶¶^ at the time of abortion for 2018, approximately three fourths (77.7%) of abortions were performed at ≤9 weeks’ gestation, and nearly all (92.2%) were performed at ≤13 weeks’ gestation (Table 9). Fewer abortions were performed at 14–20 weeks’ gestation (6.9%) or at ≥21 weeks’ gestation (1.0%). Among the 34 reporting areas that provided data every year on gestational age for 2009–2018, the percentage of abortions performed at ≤13 weeks’ gestation changed negligibly, from 91.8% to 91.5% (Table 10). However, within this gestational age range, a shift occurred toward earlier gestational ages, with the percentage of abortions performed at ≤6 weeks’ gestation increasing 8% and the percentage of abortions performed at 7–9 weeks’ and 10–13 weeks’ gestation decreasing 2% and 14%, respectively. During 2009–2018, abortions performed at >13 weeks’ gestation accounted for ≤9.0% of abortions. Among the 45 areas that reported by method type for 2018 and included medical abortion on their reporting form, 52.1% of abortions were surgical abortions at ≤13 weeks’ gestation, 38.6% were early medical abortions (a nonsurgical abortion at ≤9 weeks’ gestation), 7.8% were surgical abortions at >13 weeks’ gestation, and 1.4% were medical abortions at >9 weeks’ gestation; other methods, including intrauterine instillation and hysterectomy/hysterotomy, were both uncommon (<0.1%) (Table 11). Among the 37 reporting areas*** that included medical abortion on their reporting form and provided these data for the relevant years of comparison, use of early medical abortion increased 9% from 2017 to 2018 (from 34.7% of abortions to 37.7%) and 120% from 2009 to 2018 (from 17.1% of abortions to 37.7%). Increases in early medical abortion occurred both from 2009 to 2013 (from 17.1% of abortions to 22.7% [33% increase]) and from 2014 to 2018 (from 23.3% of abortions to 37.7% [62% increase]).

**TABLE 9:** Reported abortions, by known weeks of gestation* and reporting area of occurrence — selected reporting areas,^†^ United States, 2018

State/Area	Weeks of gestation							Total abortions reported by known gestational age
	≤6	7–9	10–13	14–15	16–17	18–20	≥21	
	No. (%)^§^	No. (%)	No. (%)	No. (%)	No. (%)	No. (%)	No. (%)	No. (% of all reported abortions)^¶^
Alabama	1,135 (17.5)	3,219 (49.7)	1,382 (21.3)	326 (5.0)	169 (2.6)	175 (2.7)	77 (1.2)	**6,483 (100.0)**
Alaska	323 (25.2)	653 (51.0)	252 (19.7)	52 (4.1)	—^††^	—	0 (0.0)	**1,281 (99.8)**
Arizona	3,685 (29.6)	5,704 (45.9)	1,921 (15.4)	469 (3.8)	254 (2.0)	266 (2.1)	139 (1.1)	**12,438 (100.0)**
Arkansas**	639 (20.8)	1,506 (49.1)	524 (17.1)	120 (3.9)	110 (3.6)	149 (4.9)	21 (0.7)	**3,069 (100.0)**
Colorado	3,000 (33.4)	3,986 (44.4)	1,172 (13.1)	218 (2.4)	161 (1.8)	109 (1.2)	323 (3.6)	**8,969 (99.9)**
Connecticut	4,196 (46.3)	3,257 (36.0)	952 (10.5)	257 (2.8)	158 (1.7)	147 (1.6)	89 (1.0)	**9,056 (97.4)**
Delaware	314 (18.1)	853 (49.1)	437 (25.1)	94 (5.4)	17 (1.0)	14 (0.8)	9 (0.5)	**1,738 (99.9)**
Florida	50,863 (72.4)	11,934 (17.0)	4,885 (7.0)	983 (1.4)	662 (0.9)	697 (1.0)	215 (0.3)	**70,239 (100.0)**
Georgia	13,352 (39.4)	12,805 (37.8)	4,892 (14.4)	1,009 (3.0)	775 (2.3)	826 (2.4)	259 (0.8)	**33,918 (100.0)**
Hawaii	580 (27.4)	955 (45.1)	366 (17.3)	75 (3.5)	56 (2.6)	64 (3.0)	23 (1.1)	**2,119 (99.9)**
Idaho	310 (24.7)	650 (51.8)	251 (20.0)	36 (2.9)	8 (0.6)	0 (0.0)	0 (0.0)	**1,255 (99.8)**
Indiana	1,401 (17.4)	4,619 (57.5)	1,983 (24.7)	7 (0.1)	7 (0.1)	9 (0.1)	9 (0.1)	**8,035 (100.0)**
Iowa	1,298 (45.6)	1,094 (38.4)	296 (10.4)	62 (2.2)	45 (1.6)	47 (1.6)	7 (0.2)	**2,849 (100.0)**
Kansas	2,829 (40.6)	2,680 (38.4)	983 (14.1)	192 (2.8)	121 (1.7)	142 (2.0)	25 (0.4)	**6,972 (100.0)**
Kentucky	1,160 (36.2)	1,250 (39.0)	487 (15.2)	126 (3.9)	64 (2.0)	85 (2.7)	31 (1.0)	**3,203 (100.0)**
Louisiana	2,663 (32.9)	3,525 (43.6)	1,405 (17.4)	322 (4.0)	139 (1.7)	40 (0.5)	0 (0.0)	**8,094 (100.0)**
Maine	622 (31.9)	907 (46.5)	305 (15.6)	52 (2.7)	36 (1.8)	27 (1.4)	0 (0.0)	**1,949 (100.0)**
Michigan	8,502 (31.8)	11,348 (42.5)	4,105 (15.4)	1,112 (4.2)	680 (2.5)	583 (2.2)	365 (1.4)	**26,695 (99.9)**
Minnesota	4,135 (42.4)	3,463 (35.5)	1,246 (12.8)	336 (3.4)	203 (2.1)	210 (2.2)	165 (1.7)	**9,758 (98.5)**
Mississippi	892 (29.7)	1,366 (45.5)	544 (18.1)	177 (5.9)	22 (0.7)	—	—	**3,005 (100.0)**
Missouri	267 (9.2)	1,302 (44.7)	814 (28.0)	191 (6.6)	146 (5.0)	133 (4.6)	57 (2.0)	**2,910 (100.0)**
Montana	576 (34.5)	707 (42.3)	256 (15.3)	55 (3.3)	33 (2.0)	34 (2.0)	10 (0.6)	**1,671 (99.8)**
Nebraska	945 (45.5)	691 (33.3)	317 (15.3)	71 (3.4)	30 (1.4)	21 (1.0)	0 (0.0)	**2,075 (99.9)**
Nevada	3,379 (38.7)	3,650 (41.8)	1,114 (12.7)	285 (3.3)	161 (1.8)	114 (1.3)	39 (0.4)	**8,742 (99.1)**
New Jersey^§§^	9,062 (40.2)	7,674 (34.0)	2,936 (13.0)	1,026 (4.6)	693 (3.1)	595 (2.6)	552 (2.4)	**22,538 (98.3)**
New Mexico	1,654 (46.2)	940 (26.2)	370 (10.3)	105 (2.9)	68 (1.9)	111 (3.1)	335 (9.3)	**3,583 (93.1)**
New York	24,546 (33.1)	30,973 (41.8)	11,713 (15.8)	2,370 (3.2)	1,490 (2.0)	1,515 (2.0)	1,507 (2.0)	**74,114 (95.7)**
New York City	21,637 (43.6)	17,714 (35.7)	5,813 (11.7)	1,310 (2.6)	950 (1.9)	1,083 (2.2)	1,171 (2.4)	**49,678 (99.8)**
New York State	2,909 (11.9)	13,259 (54.3)	5,900 (24.1)	1,060 (4.3)	540 (2.2)	432 (1.8)	336 (1.4)	**24,436 (88.3)**
North Carolina	9,029 (33.0)	11,675 (42.7)	4,458 (16.3)	1,001 (3.7)	705 (2.6)	450 (1.6)	7 (0.0)	**27,325 (99.1)**
North Dakota	390 (34.2)	496 (43.5)	191 (16.7)	54 (4.7)	9 (0.8)	—	—	**1,141 (100.0)**
Ohio	4,806 (23.5)	8,939 (43.8)	4,356 (21.3)	994 (4.9)	590 (2.9)	634 (3.1)	106 (0.5)	**20,425 (100.0)**
Oklahoma	2,369 (47.8)	1,671 (33.7)	621 (12.5)	120 (2.4)	84 (1.7)	72 (1.5)	16 (0.3)	**4,953 (99.3)**
Oregon	3,777 (43.5)	3,204 (36.9)	1,066 (12.3)	217 (2.5)	117 (1.3)	153 (1.8)	153 (1.8)	**8,687 (99.5)**
South Carolina**	960 (20.7)	1,635 (35.2)	1,750 (37.7)	284 (6.1)	—	—	8 (0.2)	**4,646 (100.0)**
South Dakota	44 (11.6)	232 (61.2)	100 (26.4)	—	0 (0.0)	—	0 (0.0)	**379 (99.2)**
Tennessee	1,680 (15.6)	5,582 (51.7)	2,519 (23.3)	607 (5.6)	280 (2.6)	120 (1.1)	9 (0.1)	**10,797 (99.2)**
Texas**	21,299 (38.6)	21,635 (39.2)	8,124 (14.7)	1,930 (3.5)	1,083 (2.0)	813 (1.5)	256 (0.5)	**55,140 (100.0)**
Utah	1,103 (35.8)	1,274 (41.3)	446 (14.5)	97 (3.1)	62 (2.0)	58 (1.9)	42 (1.4)	**3,082 (100.0)**
Vermont	509 (42.3)	471 (39.1)	131 (10.9)	33 (2.7)	15 (1.2)	25 (2.1)	20 (1.7)	**1,204 (100.0)**
Virginia	8,783 (53.6)	5,228 (31.9)	1,935 (11.8)	66 (0.4)	91 (0.6)	203 (1.2)	93 (0.6)	**16,399 (99.5)**
Washington	7,310 (42.5)	6,637 (38.5)	1,960 (11.4)	399 (2.3)	260 (1.5)	283 (1.6)	370 (2.1)	**17,219 (99.7)**
West Virginia	383 (25.4)	669 (44.4)	289 (19.2)	126 (8.4)	34 (2.3)	—	—	**1,507 (100.0)**
**Total**	**204,770 (40.2)**	**191,059 (37.5)**	**73,854 (14.5)**	**16,058 (3.2)**	**9,644 (1.9)**	**8,936 (1.8)**	**5,341 (1.0)**	**509,662 (99.0)^¶¶^**

* Gestational age based on clinician’s estimate (Alabama, Alaska, Arizona, Connecticut, Colorado, Delaware, Florida, Georgia, Hawaii, Idaho, Indiana, Iowa, Kansas, Kentucky, Louisiana, Maine, Michigan, Minnesota, Mississippi, Missouri, Montana, Nebraska, Nevada, New Jersey, New Mexico, New York City, North Carolina, North Dakota, Ohio, Oregon, Rhode Island, South Dakota, Tennessee, Vermont, Washington, and West Virginia); gestational age calculated from the last normal menstrual period (Oklahoma and Utah); clinician’s estimate of gestation based on estimated date of conception (Virginia); probable postfertilization age (Arkansas, South Carolina, and Texas).

^†^ Data from 42 reporting areas; excludes 10 areas (California, District of Columbia, Illinois, Maryland, Massachusetts, New Hampshire, Pennsylvania, Rhode Island, Wisconsin, and Wyoming) that did not report, did not report by gestational age, or did not meet reporting standards.

^§^ Percentages for the individual component categories might not add to 100% because of rounding.

^¶^ Percentage is calculated as the number of abortions reported by known gestational age divided by the sum of abortions reported by known and unknown gestational age. Values ≥99.95% are rounded to 100.0%.

^††^ Cells with a value in the range of 1–4 or cells that would allow for calculation of these small values have been suppressed.

** Two weeks were added to the probable postfertilization age to provide a corresponding measure to gestational age based on the clinician’s estimate.

^§§^ Reporting to the central health agency is not required. Data are requested from hospitals and licensed ambulatory care facilities only.

^¶¶^ Percentage based on a total of 514,718 abortions reported among the areas that met reporting standards for gestational age.

**TABLE 10:** Reported abortions, by known weeks of gestation and year — selected reporting areas,* United States, 2009–2018

Weeks of gestation	Year										% change			
	2009	2010	2011	2012	2013	2014	2015	2016	2017	2018	2009 to 2013	2014 to 2018	2017 to 2018	2009 to 2018
**≤13 weeks’ gestation (%)**	**91.8**	**91.9**	**91.5**	**91.4**	**91.6**	**91.0**	**91.0**	**91.0**	**91.1**	**91.5**	**−0.2**	**0.5**	**0.4**	**−0.3**
≤6	33.6	34.7	34.3	35.1	34.7	33.8	34.3	34.2	35.1	36.2	3.3	7.1	3.1	7.7
7–9	40.6	40.1	40.1	39.4	39.9	40.0	40.0	40.3	40.4	40.0	−1.7	0.0	−1.0	−1.5
10–13	17.6	17.0	17.1	16.9	17.0	17.2	16.7	16.4	15.7	15.2	−3.4	−11.6	−3.2	−13.6
**>13 weeks’ gestation (%)**	**8.2**	**8.1**	**8.5**	**8.6**	**8.4**	**9.0**	**9.0**	**9.0**	**8.9**	**8.5**	**2.4**	**–5.6**	**–4.5**	**3.7**
14–15	3.3	3.3	3.4	3.5	3.4	3.5	3.5	3.6	3.4	3.4	3.0	−2.9	0.0	3.0
16–17	1.8	1.8	1.9	1.9	1.9	2.2	2.1	2.1	2.2	2.1	5.6	−4.5	−4.5	16.7
18–20	1.8	1.8	1.9	1.9	1.8	1.9	2.0	2.0	2.0	1.9	0.0	0.0	−5.0	5.6
≥21	1.3	1.2	1.4	1.3	1.3	1.3	1.3	1.3	1.3	1.2	0.0	−7.7	−7.7	−7.7
**Total (no.)**	**519,164**	**508,841**	**481,667**	**457,201**	**435,881**	**426,636**	**414,914**	**408,903**	**394,181**	**395,960**	**—**	**—**	**—**	**—**

* Data from 34 reporting areas; excludes 18 areas (California, Connecticut, Delaware, District of Columbia, Florida, Illinois, Maine, Maryland, Massachusetts, Mississippi, Nebraska, New Hampshire, New York State, Pennsylvania, Rhode Island, Vermont, Wisconsin, and Wyoming) that did not report, did not report by gestational age, or did not meet reporting standards for ≥1 year. By year, these reporting areas represent 78%–98% of the abortions reported by gestational age to CDC during 2009–2018.

**TABLE 11:** Reported abortions, by known method type and reporting area of occurrence — selected reporting areas,* United States, 2018

State/Area	Surgical^†^			Medical			Intrauterine instillation^§^	Hysterectomy/ Hysterotomy	Total abortions reported by known method type
	Surgical, ≤13 weeks’ gestation	Surgical, >13 weeks’ gestation	Surgical, unknown gestational age	Medical, ≤9 weeks’ gestation	Medical, >9 weeks’ gestation	Medical, unknown gestational age			
	No. (%)^¶^	No. (%)	No. (%)	No. (%)	No. (%)	No. (%)	No. (%)	No. (%)	No. (% of all reported abortions)**
Alabama	3,719 (57.4)	735 (11.3)	0 (0.0)	1,967 (30.4)	53 (0.8)	—^††^	0 (0.0)	—	**6,478 (99.9)**
Alaska	863 (67.3)	52 (4.1)	—	362 (28.2)	—	0 (0.0)	—	0 (0.0)	**1,283 (100.0)**
Arizona	6,345 (51.0)	972 (7.8)	0 (0.0)	4,890 (39.3)	112 (0.9)	0 (0.0)	112 (0.9)	0 (0.0)	**12,431 (99.9)**
Arkansas^§§^	1,691 (55.1)	399 (13.0)	0 (0.0)	920 (30.0)	59 (1.9)	0 (0.0)	0 (0.0)	0 (0.0)	**3,069 (100.0)**
Colorado	3,134 (38.3)	425 (5.2)	—	4,519 (55.2)	109 (1.3)	—	—	0 (0.0)	**8,193 (91.3)**
Connecticut	4,460 (48.0)	648 (7.0)	169 (1.8)	3,923 (42.2)	23 (0.2)	69 (0.7)	—	—	**9,294 (100.0)**
Delaware	776 (44.7)	129 (7.4)	0 (0.0)	781 (45.0)	49 (2.8)	—	0 (0.0)	—	**1,737 (99.8)**
District of Columbia^¶¶^	2,520 (53.0)	511 (10.8)	0 (0.0)	1,701 (35.8)	19 (0.4)	0 (0.0)	0 (0.0)	0 (0.0)	**4,751 (99.6)**
Florida	33,565 (50.1)	2,438 (3.6)	0 (0.0)	30,567 (45.7)	356 (0.5)	0 (0.0)	0 (0.0)	5 (0.0)	**66,931 (95.3)**
Georgia	16,418 (48.6)	2,864 (8.5)	0 (0.0)	14,328 (42.4)	204 (0.6)	0 (0.0)	0 (0.0)	0 (0.0)	**33,814 (99.7)**
Hawaii	1,154 (54.4)	218 (10.3)	—	744 (35.1)	—	0 (0.0)	0 (0.0)	0 (0.0)	**2,121 (100.0)**
Idaho	694 (55.3)	44 (3.5)	—	509 (40.6)	5 (0.4)	—	0 (0.0)	0 (0.0)	**1,254 (99.8)**
Indiana	4,709 (58.6)	31 (0.4)	—	3,229 (40.2)	66 (0.8)	—	0 (0.0)	0 (0.0)	**8,037 (100.0)**
Iowa	748 (26.3)	155 (5.4)	0 (0.0)	1,896 (66.6)	49 (1.7)	0 (0.0)	0 (0.0)	0 (0.0)	**2,848 (100.0)**
Kansas	2,205 (31.6)	472 (6.8)	0 (0.0)	4,240 (60.8)	53 (0.8)	0 (0.0)	0 (0.0)	0 (0.0)	**6,970 (100.0)**
Kentucky	1,356 (42.3)	297 (9.3)	—	1,540 (48.1)	9 (0.3)	0 (0.0)	—	0 (0.0)	**3,203 (100.0)**
Maine	936 (48.0)	112 (5.7)	0 (0.0)	879 (45.1)	22 (1.1)	0 (0.0)	0 (0.0)	0 (0.0)	**1,949 (100.0)**
Massachusetts***	NA	NA	10,826 (59.8)	NA	NA	7,257 (40.1)	8 (0.0)	0 (0.0)	**18,091 (99.1)**
Michigan	13,761 (51.6)	2,681 (10.0)	13 (0.0)	9,999 (37.5)	213 (0.8)	8 (0.0)	—	—	**26,677 (99.9)**
Minnesota	5,284 (53.3)	889 (9.0)	59 (0.6)	3,532 (35.6)	52 (0.5)	93 (0.9)	—	—	**9,910 (100.0)**
Mississippi	758 (25.2)	195 (6.5)	—	1,994 (66.4)	55 (1.8)	0 (0.0)	0 (0.0)	—	**3,004 (100.0)**
Missouri	2,039 (70.1)	509 (17.5)	0 (0.0)	340 (11.7)	19 (0.7)	0 (0.0)	—	—	**2,909 (100.0)**
Montana	606 (36.2)	132 (7.9)	—	912 (54.5)	19 (1.1)	—	0 (0.0)	0 (0.0)	**1,672 (99.9)**
Nebraska	730 (35.1)	121 (5.8)	—	1,211 (58.3)	13 (0.6)	—	0 (0.0)	0 (0.0)	**2,078 (100.0)**
Nevada	5,252 (61.4)	583 (6.8)	41 (0.5)	2,639 (30.9)	14 (0.2)	21 (0.2)	—	—	**8,553 (97.0)**
New Jersey^†††^	12,593 (54.9)	2,682 (11.7)	219 (1.0)	6,891 (30.0)	367 (1.6)	179 (0.8)	—	—	**22,933 (100.0)**
New York	43,279 (57.3)	6,243 (8.3)	1,141 (1.5)	21,472 (28.4)	1,887 (2.5)	1,443 (1.9)	72 (0.1)	15 (0.0)	**75,552 (97.6)**
New York City	31,443 (63.4)	4,295 (8.7)	24 (0.0)	13,390 (27.0)	438 (0.9)	8 (0.0)	18 (0.0)	15 (0.0)	**49,631 (99.7)**
New York State	11,836 (45.7)	1,948 (7.5)	1,117 (4.3)	8,082 (31.2)	1,449 (5.6)	1,435 (5.5)	54 (0.2)	0 (0.0)	**25,921 (93.6)**
North Carolina	12,241 (47.1)	2,030 (7.8)	48 (0.2)	11,410 (43.9)	200 (0.8)	41 (0.2)	—	—	**25,972 (94.2)**
North Dakota	762 (66.8)	63 (5.5)	0 (0.0)	307 (26.9)	9 (0.8)	0 (0.0)	0 (0.0)	0 (0.0)	**1,141 (100.0)**
Ohio	11,940 (58.5)	2,300 (11.3)	—	6,103 (29.9)	80 (0.4)	0 (0.0)	0 (0.0)	—	**20,425 (100.0)**
Oklahoma	2,021 (40.8)	288 (5.8)	17 (0.3)	2,587 (52.2)	19 (0.4)	20 (0.4)	0 (0.0)	0 (0.0)	**4,952 (99.2)**
Oregon	3,848 (44.1)	595 (6.8)	17 (0.2)	4,137 (47.4)	100 (1.1)	31 (0.4)	—	—	**8,731 (100.0)**
Pennsylvania^§§§^	14,056 (46.3)	3,840 (12.6)	—	10,564 (34.8)	1,902 (6.3)	0 (0.0)	0 (0.0)	—	**30,363 (100.0)**
Rhode Island	1,071 (41.8)	185 (7.2)	—	499 (19.5)	5 (0.2)	800 (31.2)	—	0 (0.0)	**2,562 (90.9)**
South Carolina^§§^	1,772 (38.1)	290 (6.2)	0 (0.0)	1,919 (41.3)	665 (14.3)	0 (0.0)	0 (0.0)	0 (0.0)	**4,646 (100.0)**
South Dakota	243 (63.6)	—	—	129 (33.8)	5 (1.3)	—	0 (0.0)	0 (0.0)	**382 (100.0)**
Tennessee	4,702 (43.5)	984 (9.1)	22 (0.2)	4,915 (45.5)	124 (1.1)	43 (0.4)	—	—	**10,808 (99.3)**
Texas^§§^	31,966 (58.0)	4,033 (7.3)	0 (0.0)	18,891 (34.3)	243 (0.4)	0 (0.0)	—	—	**55,138 (100.0)**
Utah	1,552 (51.3)	230 (7.6)	0 (0.0)	1,209 (40.0)	32 (1.1)	0 (0.0)	0 (0.0)	0 (0.0)	**3,023 (98.1)**
Vermont	436 (36.2)	86 (7.1)	0 (0.0)	670 (55.7)	11 (0.9)	0 (0.0)	0 (0.0)	0 (0.0)	**1,203 (99.9)**
Virginia	10,241 (62.4)	433 (2.6)	46 (0.3)	5,647 (34.4)	38 (0.2)	—	—	0 (0.0)	**16,407 (99.6)**
Washington	7,892 (45.8)	1,304 (7.6)	20 (0.1)	7,963 (46.2)	46 (0.3)	25 (0.1)	0 (0.0)	0 (0.0)	**17,250 (99.9)**
West Virginia	852 (56.5)	161 (10.7)	0 (0.0)	473 (31.4)	21 (1.4)	0 (0.0)	0 (0.0)	0 (0.0)	**1,507 (100.0)**
Wisconsin***^,^**^¶¶¶^**	NA	NA	4,302 (71.2)	NA	NA	1,739 (28.8)	—	—	** 6,042 (100.0)**
**Total**	**289,931 (52.1)**	**43,576 (7.8)**	—****	**214,779 (38.6)**	**7,743 (1.4)**	**—^††††^**	**219 (0.0)**	**46 (0.0)**	**556,294 (98.5)^§§§§^**

**Abbreviation:** NA = not available.

* Data from 45 reporting areas; excludes seven reporting areas (California, Illinois, Louisiana, Maryland, New Hampshire, New Mexico, and Wyoming) that did not report, did not report by method type, or did not meet reporting standards. Areas reporting by method type with unknown gestational age are included.

^†^ Includes aspiration curettage, suction curettage, manual vacuum aspiration, menstrual extraction, sharp curettage, and dilation and evacuation procedures.

^§^ Intrauterine instillations reported at ≤12 weeks’ gestation were considered as unknown for method type.

^¶^ Percentages for the individual component categories might not add to 100% because of rounding.

** Percentage is calculated as the number of abortions reported by known method type divided by the sum of abortions reported by known and unknown method type. Values ≥99.95% are rounded to 100.0%.

^††^ Cells with a value in the range of 1–4 or cells that would allow for calculation of these small values have been suppressed.

^§§^ Two weeks were added to the probable postfertilization age to provide a corresponding measure to gestational age based on the clinician’s estimate.

^¶¶^ Reporting to the central health agency is not required.

*** Numbers for surgical procedures at ≤13 weeks versus >13 weeks and for medical abortions at ≤9 weeks versus >9 weeks are not presented because gestational age data were not provided by method type.

^†††^ Reporting to the central health agency is not required. Data are requested from hospitals and licensed ambulatory care facilities only.

^§§§^ Gestational age reported in this table as ≤9 weeks includes ≤8 weeks; >9 weeks includes ≥9 weeks; ≤13 weeks includes ≤12 weeks; >13 weeks includes ≥13 weeks.

^¶¶¶^ Includes residents only. Wisconsin reports as surgical, unspecified and does not differentiate surgical procedures from hysterectomy/hysterotomy. All abortions were reported as surgical or chemically induced. For this report, all surgical abortions were classified as surgical and all chemical abortions as medical.

**** For the total only, surgical abortions reported without a gestational age were distributed among the surgical abortion categories according to the distribution of surgical abortions at known gestational age.

^††††^ For the total only, medical abortions reported without a gestational age were distributed among the medical abortion categories according to the distribution of medical abortions at known gestational age.

^§§§§^ Percentage is based on a total of 565,024 abortions reported among the areas that met reporting standards for method type.

Among the 40 areas that reported abortions categorized by individual weeks of gestation and method type, surgical abortion accounted for the largest percentage of abortions within every gestational age category, except ≤6 weeks’ gestation (Table 12). At ≤6 weeks’ gestation, surgical abortion accounted for 45.1% of abortions. Surgical abortion accounted for 55.3% of abortions at 7–9 weeks’ gestation, 93.8%–98.4% of abortions at 10–20 weeks’ gestation, and 91.9% of abortions at ≥21 weeks’ gestation. In contrast, medical abortion accounted for 54.9% of abortions at ≤6 weeks’ gestation, 44.7% of abortions at 7–9 weeks’ gestation, 6.2% of abortions at 10–13 weeks’ gestation, 1.5%–3.2% of abortions at 14–20 weeks’ gestation, and 7.2% of abortions at ≥21 weeks’ gestation. For each gestational age category (if applicable), abortions performed by intrauterine instillation or hysterectomy/hysterotomy were rare (<0.1%–0.8% of abortions).

**TABLE 12:** Reported abortions, by known weeks of gestation and method type — selected reporting areas,* United States, 2018

Method type	Weeks of gestation							Total
	≤6	7–9	10–13	14–15	16–17	18–20	≥21	
	No. (%)^†^	No. (%)	No. (%)	No. (%)	No. (%)	No. (%)	No. (%)	No. (%)
**Surgical^§^**								
≤13 weeks’ gestation	88,854 (45.1)	101,978 (55.3)	66,711 (93.8)	NA	NA	NA	NA	**257,543 (52.5)**
>13 weeks’ gestation	NA	NA	NA	15,180 (98.4)	9,086 (97.7)	8,287 (95.9)	4,272 (91.9)	**36,825 (7.5)**
**Medical^¶^**								
≤9 weeks'’ gestation	108,305 (54.9)	82,339 (44.7)	NA	NA	NA	NA	NA	**190,644 (38.9)**
>9 weeks’ gestation	NA	NA	4,399 (6.2)	232 (1.5)	168 (1.8)	274 (3.2)	334 (7.2)	**5,407 (1.1)**
**Intrauterine instillation**	—**	—	4 (0.0)	8 (0.1)	44 (0.5)	70 (0.8)	38 (0.8)	**164 (0.0)**
**Hysterectomy/Hysterotomy**	13 (0.0)	9 (0.0)	3 (0.0)	4 (0.0)	4 (0.0)	7 (0.1)	4 (0.1)	**44 (0.0)**
**Total**	**197,172 (100.0)**	**184,326 (100.0)**	**71,117 (100.0)**	**15,424 (100.0)**	**9,302 (100.0)**	**8,638 (100.0)**	**4,648 (100.0)**	**490,627 (100.0)**

**Abbreviation:** NA = not applicable.

* Data from 40 reporting areas; excludes 12 areas (California, District of Columbia, Illinois, Louisiana, Maryland, Massachusetts, New Hampshire, New Mexico, Pennsylvania, Rhode Island, Wisconsin, and Wyoming) that did not report, did not report method type by weeks of gestation, did not meet reporting standards, or did not have medical abortion as a specific category on their reporting form.

^†^ For each gestational age category, percentages of all method types might not add to 100% because of rounding.

^§^ Includes aspiration curettage, suction curettage, manual vacuum aspiration, menstrual extraction, sharp curettage, and dilation and evacuation procedures.

^¶^ The administration of medication or medications to induce an abortion; at ≤9 weeks’ gestation, typically involves the use of mifepristone and misoprostol; at >9 weeks’ gestation, typically involves the use of vaginal prostaglandins.

** Intrauterine instillations reported at ≤12 weeks’ gestation have not been included with known values.

### Weeks of Gestation by Age Group and Race/Ethnicity

In selected reporting areas, abortions that were categorized by weeks of gestation were further categorized by age and race/ethnicity (Table 13). In every subgroup for these characteristics, the largest percentage of abortions occurred at ≤9 weeks’ gestation. In 42 reporting areas, by age, 55.1% of adolescents aged <15 years and 71.5% of adolescents aged 15–19 years obtained an abortion at ≤9 weeks’ gestation, compared with ≥76.8% among age groups aged ≥20 years. Conversely, 21.7% of adolescents aged <15 years and 10.3% of adolescents aged 15–19 years obtained an abortion after 13 weeks’ gestation, compared with 7.3%–8.0% for women in older age groups. In 30 reporting areas, by race/ethnicity, 73.3% of non-Hispanic Black women obtained an abortion at ≤9 weeks’ gestation, compared with 79.6%–81.5% of women from other racial/ethnic groups. Differences in abortions after 13 weeks’ gestation across race/ethnicity were minimal (8.8% for non-Hispanic Black women, compared with 6.5%–8.1% for women in the remaining racial/ethnic groups).

**TABLE 13:** Reported abortions, by known weeks of gestation, age group, and race/ethnicity — selected reporting areas, United States, 2018

Characteristic	Weeks of gestation						
	≤6	7–9	10–13	14–15	16–17	18–20	≥21
	No. (%)	No. (%)	No. (%)	No. (%)	No. (%)	No. (%)	No. (%)
Age group (yrs)*^,†^							
<15	258 (22.9)	363 (32.2)	261 (23.2)	80 (7.1)	61 (5.4)	56 (5.0)	47 (4.2)
15–19	14,520 (32.6)	17,284 (38.9)	8,090 (18.2)	1,805 (4.1)	1,089 (2.4)	1,038 (2.3)	653 (1.5)
20–24	55,059 (38.3)	55,392 (38.5)	22,029 (15.3)	4,706 (3.3)	2,765 (1.9)	2,556 (1.8)	1,417 (1.0)
25–29	60,885 (41.0)	55,796 (37.5)	21,179 (14.2)	4,394 (3.0)	2,654 (1.8)	2,406 (1.6)	1,329 (0.9)
30–34	40,940 (42.5)	35,299 (36.7)	12,760 (13.3)	2,841 (3.0)	1,758 (1.8)	1,592 (1.7)	1,046 (1.1)
35–39	23,700 (43.3)	19,756 (36.1)	7,169 (13.1)	1,630 (3.0)	926 (1.7)	924 (1.7)	638 (1.2)
≥40	8,674 (46.9)	6,339 (34.3)	2,105 (11.4)	526 (2.8)	349 (1.9)	321 (1.7)	185 (1.0)
**Total**	**204,036 (40.2)**	**190,229 (37.5)**	**73,593 (14.5)**	**15,982 (3.1)**	**9,602 (1.9)**	**8,893 (1.8)**	**5,315 (1.0)**
Race/Ethnicity*^,§^							
Non-Hispanic							
White	56,901 (42.5)	49,684 (37.1)	17,888 (13.4)	3,796 (2.8)	2,297 (1.7)	2,214 (1.7)	1,128 (0.8)
Black	39,809 (34.7)	44,253 (38.6)	20,530 (17.9)	4,481 (3.9)	2,635 (2.3)	2,215 (1.9)	781 (0.7)
Other	11,894 (45.1)	9,210 (35.0)	3,115 (11.8)	888 (3.4)	454 (1.7)	473 (1.8)	315 (1.2)
Hispanic	33,522 (48.3)	23,022 (33.2)	8,257 (11.9)	1,948 (2.8)	1,127 (1.6)	980 (1.4)	477 (0.7)
**Total**	**142,126 (41.3)**	**126,169 (36.6)**	**49,790 (14.5)**	**11,113 (3.2)**	**6,513 (1.9)**	**5,882 (1.7)**	**2,701 (0.8)**

* Percentages for the individual component categories might not add to 100% because of rounding.

^†^ Data from 42 reporting areas; excludes 10 reporting areas (California, District of Columbia, Illinois, Maryland, Massachusetts, New Hampshire, Pennsylvania, Rhode Island, Wisconsin, and Wyoming) that did not report, did not report weeks of gestation by age, or did not meet reporting standards.

^§^ Data from 30 reporting areas; excludes 22 reporting areas (California, Colorado, District of Columbia, Georgia, Hawaii, Illinois, Iowa, Louisiana, Maine, Maryland, Massachusetts, Nebraska, New Hampshire, New Mexico, New York City, New York State, North Dakota, Oklahoma, Pennsylvania, Rhode Island, Wisconsin, and Wyoming) that did not report, did not report weeks of gestation by race/ethnicity, or did not meet reporting standards.

### Abortion Mortality

Using national PMSS data (*53*), CDC identified two abortion-related deaths for 2017, the most recent year for which data were reviewed for abortion-related deaths (Table 14). Investigation of these cases indicated that two deaths were related to legal abortion.

**TABLE 14:** Number of deaths and case-fatality rates* for abortion-related deaths reported to CDC, by type of abortion — United States, 1973–2017^†^

Year	Type of abortion				CFR per 100,000 legal abortions
	Induced		Unknown**	Total	
	Legal^§^	Illegal^¶^			
**1973–1977**					**2.09**
1973	25	19	3	**47**	
1974	26	6	1	**33**	
1975	29	4	1	**34**	
1976	11	2	1	**14**	
1977	17	4	0	**21**	
**1978–1982**					**0.78**
1978	9	7	0	**16**	
1979	22	0	0	**22**	
1980	9	1	2	**12**	
1981	8	1	0	**9**	
1982	11	1	0	**12**	
**1983–1987**					**0.66**
1983	11	1	0	**12**	
1984	12	0	0	**12**	
1985	11	1	1	**13**	
1986	11	0	2	**13**	
1987	7	2	0	**9**	
**1988–1992**					**0.74**
1988	16	0	0	**16**	
1989	12	1	0	**13**	
1990	9	0	0	**9**	
1991	11	1	0	**12**	
1992	10	0	0	**10**	
**1993–1997**					**0.52**
1993	6	1	2	**9**	
1994	10	2	0	**12**	
1995	4	0	0	**4**	
1996	9	0	0	**9**	
1997	7	0	0	**7**	
**1998–2002**					**0.63**
1998	9	0	0	**9**	
1999	4	0	0	**4**	
2000	11	0	0	**11**	
2001	7	1	0	**8**	
2002	10	0	0	**10**	
**2003–2007**					**0.60**
2003	10	0	0	**10**	
2004	7	1	0	**8**	
2005	7	0	0	**7**	
2006	7	0	0	**7**	
2007	6	0	0	**6**	
**2008–2012**					**0.65**
2008	12	0	0	**12**	
2009	8	0	0	**8**	
2010	10	0	0	**10**	
2011	2	0	0	**2**	
2012	4	0	0	**4**	
**2013–2017**					**0.44**
2013	4	0	0	**4**	
2014	6	0	0	**6**	
2015	2	0	1	**3**	
2016	6	1	1	**8**	
2017	2	0	0	**2**	

**Abbreviation:** CFR = case-fatality rate.

* Number of legal induced abortion-related deaths per 100,000 reported legal induced abortions. Because a substantial number of legal induced abortions occurred outside reporting areas that provided data to CDC, national CFRs (i.e., number of legal induced abortion-related deaths per 100,000 reported legal induced abortions in the United States) were calculated with denominator data from the Guttmacher Institute’s national survey of abortion-providing facilities. Case-fatality rates were computed for consecutive 5-year periods during 1973–2017 because rates based on <20 cases might be unstable.

^†^ Certain numbers might differ from those in reports published previously because additional information has been supplied to CDC subsequent to publication.

^§^ An abortion is defined as legal if it was performed by a licensed clinician within the limits of state law.

^¶^ An abortion is defined as illegal if it was performed by any person other than a licensed clinician.

** Unknown whether abortion was induced or spontaneous.

The annual number of deaths related to legal induced abortion has fluctuated from year to year since 1973 (Table 14). Because of this variability and the relatively limited number of deaths related to legal induced abortions every year, national legal abortion case-fatality rates were calculated for consecutive 5-year periods during 1973–2017. The national legal induced abortion case-fatality rate for 2013–2017 was 0.44 legal induced abortion-related deaths per 100,000 reported legal abortions. This case-fatality rate was lower than the rates for the preceding 5-year periods.

## Discussion

For 2018, a total of 619,591 abortions were reported to CDC by 49 areas. Of these reporting areas, 48 submitted data every year for 2009–2018, thus providing the information necessary for consistently reporting trends. Among these 48 areas, for 2018, the abortion rate was 11.3 abortions per 1,000 women aged 15–44 years, and the abortion ratio was 189 abortions per 1,000 live births. Although the rate of reported abortions declined overall from 2009 to 2018, from 2017 to 2018, the number and rate of reported abortions increased 1%, and the abortion ratio increased 2%.

Among areas that reported data continuously by age from 2009 to 2018, women in their 20s accounted for the majority of abortions and had the highest abortion rates, whereas adolescents aged ≤19 years had the lowest abortion rates. During 2009–2018, women aged ≥40 years accounted for a relatively small proportion of reported abortions (≤3.7%). However, the abortion ratio among women aged ≥40 years continues to be higher than among women aged 25–39 years. These data underscore important age differences in abortion measures.

The adolescent abortion trends described in this report are important for monitoring progress that has been made toward reducing adolescent pregnancies in the United States. From 2009 to 2018, national birth data indicate that the birth rate for adolescents aged 15–19 years decreased 54% (*44*,*55*), and the data in this report indicate that the abortion rate for the same age group decreased 55%. These findings highlight that decreases in adolescent births in the United States have been accompanied by large decreases in adolescent abortions (*44*,*55*).

As in previous years, abortion rates and ratios differ across racial/ethnic groups. For example, in 2018, compared with non-Hispanic White women, abortion rates and ratios were 3.4 and 3.0 times higher among non-Hispanic Black women and 1.7 and 1.4 times higher among Hispanic women. Similar differences have been demonstrated in other U.S.-based research (*3*,*4*,*20*–*26*,*56*). The comparatively higher abortion rates and ratios among non-Hispanic Black women have been attributed to higher unintended pregnancy rates and a greater percentage of unintended pregnancies ending in abortion in this group (*57*). The complex factors contributing to differences to ensure equitable access to quality family planning services need to be identified (*58*,*59*).

In 2018, the majority of abortions occurred early in gestation (≤9 weeks), when the risks for complications are lowest (*60*–*63*). In addition, over the last 10 years, approximately three fourths of abortions were performed at ≤9 weeks’ gestation, and this percentage increased from 74.2% in 2009 to 76.2% in 2018. Moreover, among the areas that reported abortions at ≤13 weeks’ gestation by individual week, the distribution of abortions by gestational age continued to shift toward earlier weeks of gestation, with the percentage of early abortions performed at ≤6 weeks’ gestation increasing from 33.6% in 2009 to 36.2% in 2018.

From 2009 to 2018, the percentage of abortions performed at >13 weeks’ gestation did not change appreciably, remaining at ≤9.0%. Previous research indicates that the distribution of abortions by gestational age differs by various sociodemographic characteristics (*64*–*66*). In this report, the percentage of adolescents aged ≤19 years who obtained abortions at >13 weeks’ gestation was higher than the percentage of women in older age groups who obtained abortions. Multiple factors might influence the gestational age when abortions are performed (*56*,*60*–*63*,*65*–*69*).

The trend of obtaining abortions earlier in pregnancy has been facilitated by changes in abortion practices. Research conducted in the United States during the 1970s indicated that surgical abortion procedures performed at ≤6 weeks’ gestation, compared with 7–12 weeks’ gestation, were less likely to result in successful termination of the pregnancy (*70*). However, subsequent advances in technology (e.g., improved transvaginal ultrasonography and sensitivity of pregnancy tests) have allowed very early surgical abortions to be performed with completion rates exceeding 97% (*71*–*74*). Likewise, the development of early medical abortion regimens has allowed for abortions to be performed early in gestation, with completion rates for regimens that combine mifepristone and misoprostol reaching 96%–98% (*74*–*77*). In 2018, 77.7% of all reported abortions were ≤9 weeks’ gestation thus were eligible for early medical abortion; of these, 50.0% were reported as medical abortions. Moreover, among areas that included medical abortion on their reporting form, the percentage of all abortions performed by early medical abortion increased 120% from 2009 to 2018.

Because the annual number of deaths related to legal induced abortion is small and statistically unstable, case-fatality rates were calculated for consecutive 5-year periods during 1973–2017. The national legal induced abortion case-fatality rate for 2013–2017 was fewer than 1 per 100,000 abortions, as it was for all the previous 5-year periods since the late 1970s, demonstrating the low risk for death associated with legal induced abortion.

## Limitations

The findings in this report are subject to at least four limitations. First, because reporting to CDC is voluntary and reporting requirements vary by the individual reporting areas (*28*), CDC is unable to report the total number of abortions performed in the United States. Of the 52 areas from which CDC requested data for 2018, California, Maryland, and New Hampshire did not submit abortion data. In 2017, the most recent year for which data are available through the Guttmacher Institute’s national survey of abortion-providing facilities, abortions performed in California, Maryland, and New Hampshire accounted for approximately 19% of all abortions in the United States (*19*). In addition, the District of Columbia and New Jersey did not have abortion reporting requirements to a centralized health agency during the period covered in this report (*27*), which potentially affects the representativeness of data these jurisdictions send to CDC. Moreover, even in states that legally require clinicians to submit a report for every abortion they perform, enforcement of this requirement varies.^†††^ The accuracy of comparative data reported by the Guttmacher Institute is affected by facility response rates, the accuracy of information reported by facilities, as well as the degree to which abortion counts were estimated from nonfacility data sources (*19*).

Second, many states use abortion reporting forms that differ from the technical guidance that CDC developed in collaboration with the National Association for Public Health Statistics and Information Systems. Consequently, multiple reporting areas do not collect all variables requested by CDC (e.g., age and race/ethnicity) or do not report the data in a manner consistent with this guidance (e.g., gestational age). Missing demographic information can reduce the extent to which the statistics in this report represent women who have had abortions. Findings in this report on demographic characteristics of women seeking abortions were generally similar to previously published data from Guttmacher Institute’s national survey of abortion patients in 2014, although the percentage of abortions accounted for by non-Hispanic Black women was higher and by Hispanic women was lower as compared with data provided to CDC (*25*). Differences are likely attributable to the fact that race/ethnicity data that met CDC’s reporting standards were only reported to CDC by 31 reporting areas. Some areas that either do not report to CDC (e.g., California) or do not report race/ethnicity data (e.g., Illinois) have sufficiently large populations of minority women that the absence of data from these areas likely reduces the representativeness of CDC data for these variables. In addition, some areas collect gestational age data that are based on estimated date of conception or probable postfertilization age, which are not consistent with medical conventions for gestational age reporting. Without medical guidance on how to report these data, the validity and reliability of gestational age for these reporting areas is uncertain.

Third, abortion data are compiled and reported to CDC by the central health agency of the reporting area in which the abortion was performed rather than the reporting area in which the woman lived. Thus, the available population (*33*–*42*) and birth data (*43*–*45*), which are organized by the states in which women live, might differ from the population of women who undergo abortions in a given reporting area. This likely results in an overestimation of abortions for reporting areas in which a higher percentage of abortions are obtained by out-of-state residents and an underestimation of abortions for states where residents more frequently obtain abortions out of state. Limited abortion services, stringent regulatory requirements for obtaining an abortion, or geographic proximity to services in another state might influence where women obtain abortion services (*78*).

Finally, CDC reporting of sociodemographic characteristics of women obtaining abortions is limited to data collected on jurisdiction reporting forms. Therefore, examining additional demographic variables, (e.g., socioeconomic status) is not possible.

## Public Health Implications

Ongoing surveillance of legal induced abortion is important for several reasons. First, abortion surveillance can be used to help evaluate programs aimed at preventing unintended pregnancies. Although pregnancy intentions can be difficult to assess (*79*–*84*), abortion surveillance provides an important indicator of unintended pregnancies because up to 42% of unintended pregnancies in the United States end in abortion (*57*). Efforts to help women avoid unintended pregnancies might reduce the number of abortions (*85*,*86*). Second, routine abortion surveillance is needed to assess trends in clinical practice patterns over time. Information in this report on the number of abortions performed through different methods (e.g., medical or surgical) and at different gestational ages provides the denominator data that are necessary for analyses of the relative safety of abortion practices (*54*). Finally, information on the number of pregnancies ending in abortion is needed in conjunction with data on births and fetal losses to estimate the number of pregnancies in the United States and determine rates for various outcomes of public health importance (e.g., adolescent pregnancies) (*87*).

Approximately 18% of all pregnancies in the United States end in induced abortion (*19*). Multiple factors influence the incidence of abortion, including access to health care services and contraception (*85*,*86*,*88*,*89*); the availability of abortion providers (*8*,*11*,*16*,*90*–*93*); state regulations, such as mandatory waiting periods (*69*,*94*,*95*), parental involvement laws (*96*,*97*), and legal restrictions on abortion providers (*98*–*102*); increasing acceptance of nonmarital childbearing (*103*,*104*); and changes in the economy and the resulting impact on fertility and contraceptive use (*105*).

The most recent data available indicate that the proportion of pregnancies in the United States that were unintended decreased from 51% in 2008 to 45% during 2011–2013 (*57*). Changing patterns of contraception use might have contributed to this decrease in unintended pregnancy. Use of long-acting reversible contraception (LARC) (i.e., intrauterine devices and hormonal implants), which are the most effective reversible contraceptive methods, has recently increased among all women (*106*–*109*), and the use of contraception overall appears to be increasing among sexually active adolescents (*110*). In addition, immediate postpartum and postabortion contraception provision, especially of LARC, has been shown to decrease rapid repeat pregnancy and repeat abortions (*111*–*117*). Further, providing contraception for women at low or no cost can increase use of more effective contraceptive methods for pregnancy prevention and reduce unintended pregnancy and abortion rates (*85*,*86*,*88*,*118*–*120*). Inadequate provider reimbursement and training, insufficient client-centered counseling, lack of youth-friendly services, and low client awareness of available contraceptive methods are reported barriers to accessing contraception (*121*–*124*). Reducing these barriers might help improve access to contraception and potentially reduce the number of unintended pregnancies and the number of abortions in the United States.

## Footnotes

* Hereafter, all abortions in this report are considered to be legally induced unless stated otherwise.

† Includes dilation and curettage (aspiration curettage, suction curettage, manual vacuum aspiration, menstrual extraction, and sharp curettage) and dilation and evacuation procedures.

§ CDC collects information only on the estimated number of weeks (not days) of gestation and acknowledges the conventional use of completed weeks of gestation to describe pregnancy duration. CDC’s category ≤9 weeks’ gestation thus includes abortions up through 9 weeks and 6 days.

¶ The cutoff of ≤12 weeks was selected on the basis of the implausibility of this procedure being performed at earlier gestational ages.

** States that did not report for ≥1 year from 1998 to 2017 include Alaska (1998–2000), California (1998–2017), District of Columbia (2016), Louisiana (2005), Maryland (2007–2017), New Hampshire (1998–2017), Oklahoma (1998–1999), and West Virginia (2003–2004).

†† Comparisons do not include Wyoming, which reported <20 abortions and therefore was excluded from this and all subsequent analyses.

§§ Excludes California, Connecticut, Illinois, Maryland, New Hampshire, and Wyoming.

¶¶ Arkansas, South Carolina, and Texas collected probable postfertilization age. Two weeks were added to the probable postfertilization age to provide a corresponding measure to gestational age based on the clinician’s estimate. Virginia reported clinician’s estimate of gestational age based on conception; no modifications were made to these data.

*** Excludes Alabama, California, Delaware, Florida, Hawaii, Illinois, Louisiana, Maryland, Nevada, New Hampshire, New Mexico, Tennessee, Vermont, Wisconsin, and Wyoming

††† For 2018, data that Wyoming reported to CDC were <5% of the estimated abortions reported by the Guttmacher Institute through their 2017 national survey of abortion-providing facilities, the most recent year for which the Guttmacher Institute has published data. CDC 2018 numbers for Colorado, Connecticut, District of Columbia, Hawaii, Iowa, Louisiana, Missouri, New Jersey, New Mexico, New York (city and state combined), Rhode Island, and South Dakota were 48%–85% of the Guttmacher Institute 2017 estimates. All other areas that provided data to CDC for 2018 were ≥90% of Guttmacher Institute 2017 estimates.
