# *Erratum*: Vol. 69, No. SS-7

**DOI:** 10.15585/mmwr.mm7049a5

**Published:** 2021-12-10

In the Surveillance Summary “Abortion Surveillance — United States, 2018,” on page 5, the last sentence of the second paragraph should have read, “Overall, **0.9%** of abortions were reported to CDC with unknown residence.” On page 9, the fourth sentence of the first paragraph should have read, “Findings in this report on demographic characteristics of women seeking abortions were generally similar to previously published data from Guttmacher Institute’s national survey of abortion patients in 2014, although the percentage of abortions accounted for by non-Hispanic Black women was **lower** and by Hispanic women was **higher** as compared with data provided to CDC (*25*).”

