# *Erratum:* Vol. 69, No. SS-7

**DOI:** 10.15585/mmwr.mm7139a4

**Published:** 2022-09-30

In the Surveillance Summary “Abortion Surveillance — United States, 2018,” on page 15, in Table 2, under the column heading “Abortions obtained by out-of-state residents,” the value for New York should have read **4,743 (6.1)**.

